# Full Blood Count Trends for Colorectal Cancer Detection in Primary Care: Development and Validation of a Dynamic Prediction Model

**DOI:** 10.3390/cancers14194779

**Published:** 2022-09-29

**Authors:** Pradeep S. Virdee, Julietta Patnick, Peter Watkinson, Tim Holt, Jacqueline Birks

**Affiliations:** 1 Nuffield Department of Primary Care Health Sciences, University of Oxford, Oxford OX2 6GG, UK; 2 Nuffield Department of Population Health, University of Oxford, Oxford OX3 7LF, UK; 3 Kadoorie Centre for Critical Care Research and Education, Oxford University Hospitals NHS Trust, Oxford OX3 9DU, UK; 4 Centre for Statistics in Medicine, NDORMS, University of Oxford, Oxford OX3 7LD, UK

**Keywords:** full blood count, blood test, primary care, colorectal cancer, prediction model, joint modelling of longitudinal and time-to-event data

## Abstract

**Simple Summary:**

Colorectal cancer is the fourth most common cancer and second most common cause of cancer-death in the UK. If diagnosed and treated early-stage, when the cancer has not spread, 9 in 10 patients are alive five years later. If diagnosed at a late-stage, when the cancer has spread, this drops to 1 in 10 alive. Early detection can save lives, but more than half of colorectal cancers are diagnosed late-stage in the UK. Growing tumours often cause subtle changes in blood test results that could help with earlier detection. For example, patients diagnosed with colorectal cancer often have an increasingly lowering haemoglobin for a few years before their diagnosis, which is not seen in patients without colorectal cancer. These differences as subtle so may be difficult for doctors in primary care to spot from a series of blood tests. We developed a computer-based tool to do this. This tool checks the changes in a patient’s blood test results over the last five years to see how likely they are to have colorectal cancer. We report this tool here and describe how well it works in identifying colorectal cancer cases using blood tests performed in primary care.

**Abstract:**

Colorectal cancer has low survival rates when late-stage, so earlier detection is important. The full blood count (FBC) is a common blood test performed in primary care. Relevant trends in repeated FBCs are related to colorectal cancer presence. We developed and internally validated dynamic prediction models utilising trends for early detection. We performed a cohort study. Sex-stratified multivariate joint models included age at baseline (most recent FBC) and simultaneous trends over historical haemoglobin, mean corpuscular volume (MCV), and platelet measurements up to baseline FBC for two-year risk of diagnosis. Performance measures included the c-statistic and calibration slope. We analysed 250,716 males and 246,695 females in the development cohort and 312,444 males and 462,900 females in the validation cohort, with 0.4% of males and 0.3% of females diagnosed two years after baseline FBC. Compared to average population trends, patient-level declines in haemoglobin and MCV and rise in platelets up to baseline FBC increased risk of diagnosis in two years. C-statistic: 0.751 (males) and 0.763 (females). Calibration slope: 1.06 (males) and 1.05 (females). Our models perform well, with low miscalibration. Utilising trends could bring forward diagnoses to earlier stages and improve survival rates. External validation is now required.

## 1. Introduction

Colorectal cancer is the fourth most common type of cancer [1] and second most common cause of cancer-related death [2] in the UK. Prognosis is heavily influenced by tumour stage at diagnosis: five-year survival is 93% if diagnosed at Stage I and 10% if at Stage IV [3]. Identification at earlier stages would improve likelihood of successful treatment and reduce mortality [4]. Relying on the onset of symptoms is limited, as these are non-specific, such as abdominal pain and change in bowel habit. Current evidence indicates that symptom reporting to primary care is highest within six months prior to diagnosis, with symptom-reporting prevalance at time-points prior to 18 months comparable between cases and non-cases [5]. Symptom-based approaches, such as the QCancer Colorectal prediction model, are therefore likely to identify people with relatively late-stage disease [6]. 

The full blood count (FBC) is a blood test commonly performed in primary care [7]. We previously reported trends in the FBC test over a 10-year period, with colorectal cancer patients having on average a different trend within four years prior to diagnosis than patients without this diagnosis [8]. Our study suggested that relevant trends may appear before abnormal FBC thresholds [9,10,11,12] and referral thresholds for further cancer investigation [13,14,15] are reached and before the onset of apparent symptoms. Utilising trends might therefore facilitate earlier detection. 

The ColonFlag (previously MeScore), developed in Israel, uses machine-learning techniques to flag patients with colorectal cancer based on their age, sex, and changes in FBC results over a three-year period [16]. We externally validated the ColonFlag model using UK primary care data, demonstrating that it could discriminate high-risk from low-risk patients at 18–24 months before diagnosis (AUC = 0.78) [17]. However, the underlying methodology is non-transparent so cannot easily be incorporated into practice. 

The aims of this study were to develop and internally validate prediction models that incorporate patient-level trends in repeated FBCs from primary care to predict two-year risk of colorectal cancer and compare predictive performance to the ColonFlag model. We hypothesis that trends in FBC results over time have predictive value for colorectal cancer detection.

## 2. Methods

Study reporting follows the TRIPOD guidelines [18]. Data preparation was performed in Stata/SE V15.1 and analyses in RStudio (R V4.0.2).

### 2.1. Data

FBC data were obtained from a UK primary care database, the Clinical Practice Research Datalink (CPRD) GOLD [19], and diagnosis data from the UK National Cancer Registration and Analysis Service (NCRAS) (CPRD protocol: 14_195RMn2A2R). Data between 1 January 2000 and 14 January 2014 (data-cut date) was extracted. Clinical codes for data extraction have previously been published [8]. 

### 2.2. Study Design

**Figure 1:**
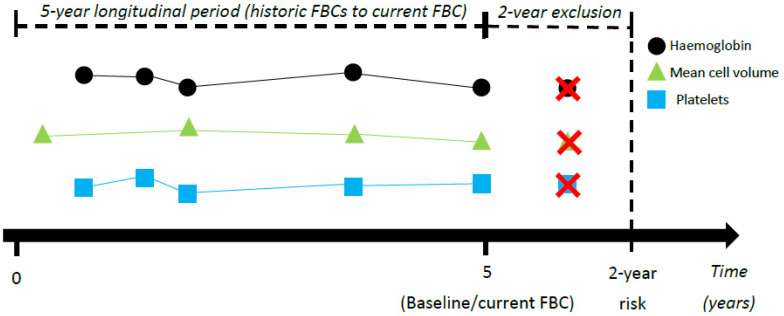
Flow of (dummy) longitudinal data for two-year risk of colorectal cancer diagnosis. Red X indicates tests that were excluded.

We performed a prospective cohort study (see Figure 1). Baseline was defined as the date of the most recent FBC available. FBCs performed before baseline were considered historic. Trends were identified using historical FBCs over a five-year period up to baseline FBC. Risk predictions are therefore made from the baseline time-point, incorporating information from historical FBCs. A five-year longitudinal period was chosen based on our previous work showing differences in trends between patients with and without a diagnosis confined to five years pre-diagnosis [8]. 

A hypothetical patient is described here to help clarify the study design. Assume the date is June 2022. A patient visits their GP, who orders a FBC blood test, performed in June 2022 and now entered the patient’s electronic GP record. This current FBC is considered baseline and corresponds to the end of the five-year longitudinal period (June 2022, time = 5). The start of the five-year longitudinal period is therefore five years earlier (June 2017, time = 0). Assume the patient already had four FBCs in the past, with the earliest measured in September 2017. The earliest is then measured at time = 0.25 years into this five-year period (i.e., three months after the start of the longitudinal period: June 2017 to September 2017). Trends over historic FBCs in this five-year period up to the current/baseline FBC (time = 0 to 5) are used to identify risk of diagnosis two years in the future following baseline FBC.

### 2.3. Participants

Patients aged at least 40 years with at least one haemoglobin, mean corpuscular volume (MCV), and platelet measurement available in their primary care record were included. Patients were excluded if registered with their primary care practice for less than one year, had a history of colorectal cancer before study entry, or not linked to the NCRAS registry. Patients with an available date of diagnosis but no indication of cancer were excluded.

For the internal validation cohort, patients with no ColonFlag score corresponding to the baseline FBC were excluded to ensure the models were compared on the same patient sample and two-year risk scores from the same FBC. The ColonFlag score was derived by Medial EarlySign and returned to us for analysis, with reasons for missing ColonFlag scores unknown.

### 2.4. Outcome

The outcome was a diagnosis of colorectal cancer in two years (+/− three months) after baseline FBC. Patients without a diagnosis were censored at the earliest of date of leaving the practice, date of death, date of diagnosis of another cancer type, 14 January 2014 (the data-cut date), or two years after their baseline FBC.

FBCs within the two years (+/− three months) proceeding diagnosis/censor date were excluded from the study (Figure 1). This two-year exclusion period ensures each patient has two years of follow-up following the resulting most recent FBC (baseline), i.e., there were no patients diagnosed/censored within two years (+/− three months) following baseline. It also ensures only data earlier than two years before diagnosis/censor date is used to identify risk to facilitate detection at a sufficiently earlier phase that clinical intervention is likely to impact prognosis. Furthermore, it reduces bias resulting from cases having more FBCs performed closer together in the run up to diagnosis than non-cases.

### 2.5. Predictors

A separate model was developed for males and females. Age at baseline FBC and trends in historic haemoglobin, MCV, and platelet measurements up to baseline FBC were included as predictors. Reasons why only these were considered are in Supplementary Methods File S1 and include our systematic review identifying these as relevant parameters [20]. We excluded FBC results outside biologically plausible ranges, which have previously been reported [8], such as negative values. 

### 2.6. Missing Data

Year of birth and sex were available for all patients and there was little (<~5%) missing haemoglobin, MCV, and platelet data among all FBCs. We therefore performed no data imputation and modelled all data as-is. A detailed account of our FBC data preparation and validation processes has previously been reported [21].

### 2.7. Sample Size

The required sample size was calculated using the *pmsampsize* package in Stata software [22]. The package is designed for conventional modelling approaches, such as logistic and Cox models, so do not provide sample sizes for joint models, although a Cox sub-model is used in the joint model. It was used here to provide an indication of the minimum sample size required.

A time-to-event outcome was used. Mean follow-up time in the available CPRD data was 3.8 years for males and 4.1 years for females. Five predictor parameters were planned for inclusion in the Cox sub-model: two fractional polynomial terms for age at baseline FBC and one term for each of the three FBC parameters. A 0.2% two-year event rate for both males and females was assumed, based on the internal validation of the QCancer Colorectal prediction [6]. The Cox-Snell R^2^ for the QCancer Colorectal models was 0.003079 for males and 0.0029112 for females, derived from the reported area under the curve (AUC) [23]. A 0.9 shrinkage factor was assumed to adjust for overfitting. Based on these estimates, 14,591 males and 15,433 females were required, with 123 and 130 events, respectively.

### 2.8. Model Development

A multivariate joint model of longitudinal and time-to-event data was developed for males and females separately. Multivariate joint models consist of two linked models: the mixed-effects sub-model (one for each FBC parameter, to model trends) and the Cox sub-model (for risk predictions) [24,25,26,27,28,29]. They are considered dynamic because they use repeated measures data to identify risk, which is updated as new measurements become available. Further details of joint models are in Supplementary Methods File S1.

In the mixed-effects sub-models, age at baseline FBC (fixed effect) was modelled using linear splines, with knots at age 60, 70, and 80 for haemoglobin, 55 for MCV, and 60 for platelets. Time to baseline FBC (fixed effect) was modelled using linear splines, with a knot at year 3 in the five-year longitudinal period for each FBC parameter. An interaction between time and age (fixed effect) was included in the haemoglobin mixed-effects model only. A random intercept for patient and random slope for time was used for each FBC parameter, with an unstructured covariance matrix to account for correlated repeated measures. In the Cox sub-model, age at baseline FBC was modelled using fractional polynomials (powers: 2, 2, determined automatically by the software). Age-adjusted patient-level FBC trends were pulled automatically from the mixed-effect sub-models and included as covariates in the Cox sub-model, as per the joint modelling framework. Further details of model development are in Supplementary Methods File S1.

The Breslow baseline survival estimate, which uses mean-centred predictors, was estimated at two years from the baseline FBC and combined with the Cox model coefficients to give absolute risk predictions.

### 2.9. Model Validation

Joint models are computationally intensive and burden on computer capacity increases when combined with big datasets. We used an advanced ‘super-computer’ to develop the model, which was still insufficient for the entire patient sample and could facilitate approximately 250,000 patients only. Therefore, the final sample was split randomly until around 250,000 remained in the development cohort for males and females separately. The remaining patients were considered an internal validation split sample cohort. 

Predictive performance was assessed in the overall internal validation cohorts and in relevant subgroups of age (10-year age bands from 40 to 90 years), number of FBCs available (from 2 to 14 FBCs, with limited sample sizes beyond 14), and time span of FBCs (6-month time bands from 0 to 5 years).

In the internal validation cohort, predictive performance of the joint models was compared to the ColonFlag model [16]. The ColonFlag uses changes in repeated FBCs measured around 15 and 33 months prior to the baseline FBC to identify a monotone score of 0–100 (0 = lowest risk, 100 = highest risk) for diagnosis. The ColonFlag score for each patient’s baseline FBC was derived by Medial EarlySign in confidence and returned to us for analysis. The ColonFlag was developed using a pooled cohort of males and females, but performance was assessed separately by sex here for comparability with the joint models. 

### 2.10. Model Performance

Performance of the joint models was assessed in the development and internal validation cohorts separately. Overall performance was assessed using Royston and Sauerbrei’s (pseudo) R_D_-squared [30] (can be very small; higher is better) and Brier score [31] (=0 indicates no difference between observed and predicted risks; lower is better). Discrimination was assessed using the c-statistic (or AUC) (conventional rule-of-thumb of ≥0.7 indicates good discrimination; higher is better) and Royston and Sauerbrei’s D-statistic [30] (higher is better). Calibration was assessed using the calibration slope (=1 indicates perfect calibration) and calibration plots. Calibration plots were derived by first categorising patients into 20 equally sized groups of predicted two-year risk and the mean of the predicted two-year risk compared with the observed two-year risk for each risk group separately. The observed two-year risk for each group was estimated using the Kaplan–Meier survival function to account for censored observations.

In the internal validation cohort, discrimination of the ColonFlag was assessed using the c-statistic. As the machine-learning algorithm does not provide a measure of absolute risk, the other performance measures could not be derived. For both the joint models and ColonFlag, the c-statistic was assessed and compared in the overall cohorts and in specified subgroups. Calibration plots for our joint models were also derived for each subgroup.

### 2.11. Diagnostic Accuracy and Risk Thresholds

For the joint models, two-year risk thresholds corresponding to various risk percentiles, from 75th to 99th percentile of predicted risk, were derived in the internal validation cohort. Diagnostic accuracy measures were calculated for each threshold of risk: sensitivity, specificity, positive predictive value (PPV), and negative predictive value (NPV). A receiver operating characteristic (ROC) curve was derived for both the joint models and ColonFlag model. 

## 3. Results

### 3.1. Summary of Patient Data

**Figure 2:**
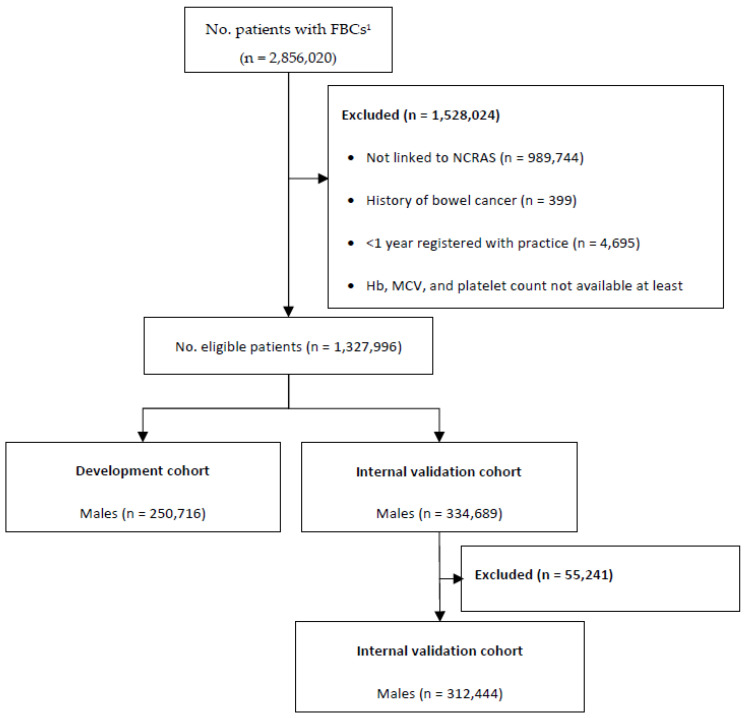
Patient flow diagram. ^1^ Number of patients available in the CPRD data extract. Abbreviations: FBC = full blood count; NCRAS = National Cancer Registration and Analysis Service; Hb = haemoglobin; MCV = mean corpuscular volume.

We identified 585,405 eligible males and 742,591 eligible females (Figure 2). There were 42.8% (n = 250,716) males and 33.2% (n = 246,695) females assigned to the development cohort, with 865 (0.4%) and 677 (0.3%) diagnosed with colorectal cancer two years (+/− three months) after their baseline FBC, respectively. 

There were 334,689 males and 495,896 females assigned to the internal validation cohort. A further 22,245 males and 32,996 females were excluded, as they had no ColonFlag score corresponding to their baseline FBC. This resulted in 312,444 males and 462,900 females included, with 1040 (0.4%) and 1200 (0.3%) diagnosed with colorectal cancer two years after their baseline FBC, respectively.

A summary of patient characteristics is in Table 1 and a summary of FBC and follow-up data is in Table S1 (development cohort) and Table S2 (internal validation cohort). On average, patients diagnosed were approximately 10 years older than patients not diagnosed. Summary statistics of FBC and follow-up data were balanced between patients with and without colorectal cancer and the development and internal validation cohorts. A summary of cancer staging (Duke’s) is in Table S3.

### 3.2. Model Development

Among the 250,716 males in the development cohort, there were 800,355 haemoglobin, 784,968 MCV, and 786,474 platelet measurements in the five-year longitudinal period used to build the model. For the 246,695 females in the development cohort, this was 907,841 haemoglobin, 891,903 MCV, and 891,380 platelet measurements. 

Coefficients from the mixed-effects sub-models, where trends in the blood levels are identified, from the final multivariate joint models are provided in Table S4 (fixed effects) and Table S5 (random effects variance-covariance matrix). Hazard ratios from the Cox models, where two-year risk of diagnosis is determined, are in Table 2. The hazard ratios indicate that a patient-level decline in haemoglobin and MCV with a rise in platelet count from the average population trend (non-cases), identified from mixed-effects sub-models, increases two-year risk of diagnosis.

### 3.3. Model Performance

Model performance statistics for the development and internal validation cohorts are provided in Table 3. Performance was comparable between the development and validation cohorts. The Brier score for overall performance was close to zero, suggesting little difference between observed and predicted risks for both males and females. The c-statistic was 0.751 for males and 0.763 for females in the validation cohort, suggesting the models can discriminate high-risk from low-risk patients using only earlier data prior to two years before diagnosis. 

**Figure 3:**
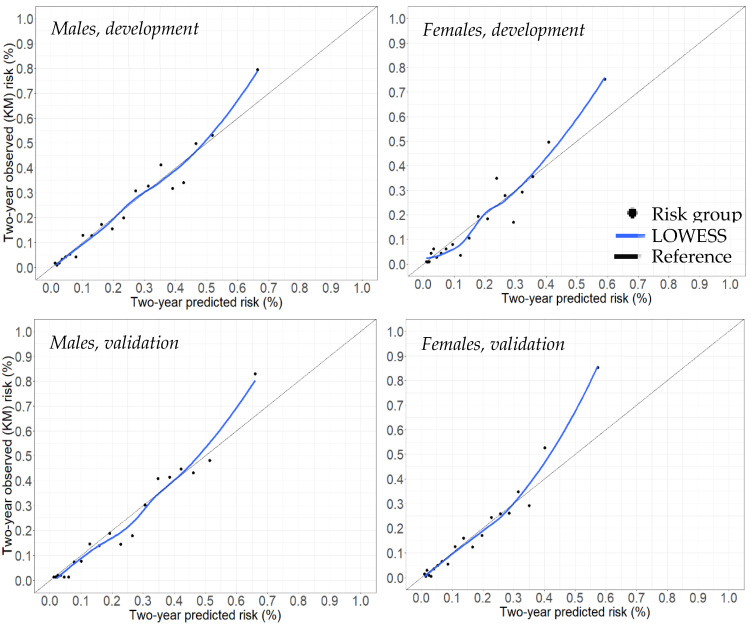
Calibration plots for the joint models. Abbreviations: KM = Kaplan–Meier.

The calibration slope was 1.06 for males and 1.05 for females in the validation cohort, suggesting the presence of little under-prediction. Calibration plots also indicate good calibration (Figure 3), with predicted and observed two-year risk matching closely, hovering over the reference (y = x) line for perfect calibration. The curves diverge slightly from the reference line for the 20th risk percentile group, suggesting a slight under-prediction in the highest risk group. 

### 3.4. Performance in Subgroups (Validation Cohort)

A summary of patient data (FBC and follow-up data) is provided by age group in Table S6. The median number of FBCs and follow-up time and amount of missing data was similar between patients with and without a diagnosis per age group. The c-statistic increased as females grew older and was more variable for males (Figure S1). Calibration plots by age group are in Figure S2 for the joint models. The models were well calibrated in all age groups, although under-predicted risk of diagnosis in males aged 90+ years at baseline FBC, which may be due to the small number of events in the subgroup (n = 21 diagnosed, Table S6) or other health reasons.

A summary of patient data (age, FBC data, and follow-up data) is provided by number of FBCs in Table S7. Patients diagnosed were on average approximately 10 years older at baseline FBC than patients not diagnosed. The median duration of the five-year longitudinal period, follow-up time, and amount of missing data were similar between patients with and without a diagnosis. Unexpectedly, the c-statistic decreased as the number of tests increased (Figure S3), which may be because FBCs are more common in older age groups (Table S6), where there is a higher likelihood of comorbidity. Additionally, the sample size was small among patients with higher numbers of FBCs available, producing wide confidence intervals. To minimise the influence of age on the c-statistic, these are provided as an example for males aged 70–89 years at baseline FBC, as this age group had the largest sample size and number of events (Figure S3). In this age group, the c-statistic increased as the number of FBCs increased, as expected. However, the small sample size for each group produced wide confidence intervals. Calibration plots by number of FBCs are given in Figure S4 for the joint models. The models were generally well calibrated regardless of the number of FBCs used to identify two-year risk of diagnosis, but under-predicted risk for males with ≥ 8 FBCs, an effect not observed in females. This was checked in the development cohort, where this under-prediction reduced substantially (Figure S4), suggesting that the high event-rate observed in these subgroups may be specific to this internal validation cohort. 

A summary of patient data (age and FBC data) is provided by time span of FBCs in Table S8. Patient data were comparable between patients with and without a diagnosis, although cases were on average approximately 10 years older at baseline FBC than patients not diagnosed. The c-statistic was similar regardless of the time span of FBCs, although decreased slightly for males whose FBCs spanned a three-year period or longer (Figure S5). Calibration plots by time span of FBCs are in Figure S6 for the joint models. The models were well calibrated regardless of how spread apart the FBCs were.

### 3.5. Diagnostic Accuracy and Risk Thresholds (Validation Cohort)

**Figure 4:**
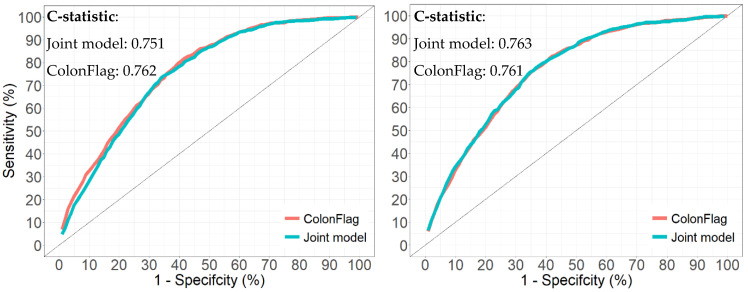
ROC curves for the joint models and ColonFlag for males (**left**) and females (**right**) (validation cohort).

Diagnostic test accuracy measures are in Table 4. The lowest risk percentile, 75%, corresponded to a risk cut-off of 0.3670% for males and 0.2767% for females, with 57.69% and 59.17% sensitivity and 75.11% and 75.09% specificity, respectively. The highest risk percentile, 99%, corresponded to a risk cut-off of 0.7232% for males and 0.6446% for females, with 4.71% and 6.25% sensitivity, respectively, and 99.01% specificity for both males and females. The NPV ranged 99.68–99.86%, indicating a high proportion of patients with low predicted risk without an observed diagnosis. ROC curves for the joint models are in Figure 4. 

### 3.6. Comparison to the ColonFlag (Validation Cohort)

ROC curves and c-statistics (or AUC) for the joint models and ColonFlag are in Figure 4. ROC curves were superimposed for the two models, indicating similar diagnostic ability for two-year risk. Additionally, the c-statistic was comparable in males (joint model 0.751, ColonFlag 0.762) and females (joint model 0.763, ColonFlag 0.761), indicating similar discriminative ability between the two models. In all subgroups assessed, the c-statistic was similar between the joint models and ColonFlag model, although slightly higher for the ColonFlag for most age groups in males.

## 4. Discussion

### 4.1. Summary of Main Findings

Many FBC parameters change over time due to colorectal cancer [8]. The opportunity to use these changes to detect colorectal cancer is currently missed in practice because blood levels often remain in the normal reference range (so are not flagged as abnormal) and clinicians usually only assess the most recent FBC. We utilised these relevant changes in a dynamic clinical risk prediction model, harnessing repeated FBC measures to identify two-year risk of diagnosis. A patient-level decline in haemoglobin and MCV and rise in platelet count from the average population trends increased the risk of diagnosis in two years. Our multivariate models have good predictive performance and calibration, with only adjustments for age, sex, and trends in haemoglobin, MCV, and platelet count earlier than two years before diagnosis. 

Performance of the joint models was good in the internal validation cohort and comparable to the development cohort, suggesting little-to-no optimistic performance [32,33,34,35,36]. For example, the NPV for the joint models was around 99.7% or above for almost all thresholds, suggesting the models have high performance in identifying diagnosis-free patients and could therefore help avoid unnecessary referrals in practice. This would ultimately reduce burden on healthcare services, such as staff, time, and cost. Performance of the joint models was good for almost all subgroups of age at baseline FBC and by number of FBCs used to identify risk. Performance in subgroups should be assessed in larger samples, which will be considered as future work. 

The joint models and ColonFlag model performed very similarly for two-year risk, both overall and in subgroups. This was expected, as they ultimately use the same data (age, sex, changes over time in FBC levels) to identify risk. Discrimination was only slightly better for the ColonFlag, which may be because it uses up to all 20 FBC parameters to identify risk [16], whereas our joint models use only haemoglobin, MCV, and platelets. However, discrimination remained very similar regardless, suggesting these additional parameters may not improve risk estimation much. The relative simplicity of our models lends them more readily to adoption and embedding within electronic health record systems, to facilitate the identification and flagging of cancer risk during routine care.

### 4.2. Comparison with Existing Literature

Many prediction models for colorectal (bowel) cancer exist. We identified 13 models in our systematic review that use some FBC data to identify risk of bowel cancer [20]. All but one (ColonFlag by Kinar 2016 [16]) are static models, meaning they use data from one baseline time-point. This includes the most commonly used colorectal cancer prediction model in the UK, the QCancer Colorectal model by Hippisley-Cox 2013 [6]. QCancer Colorectal relies on symptoms to identify two-year risk of diagnosis, but recent studies identified that symptoms are commonly reported close to the time of diagnosis [5,37], suggesting the model may not perform well for early detection. Our joint models are an improvement on these static models, including QCancer Colorectal, because they use repeated measures from a single patient to provide a more individualised risk prediction. Additionally, compared to the QCancer Colorectal model, our joint models rely only on earlier data recorded prior to two years before diagnosis and have good performance, suggesting they could predate symptoms and facilitate earlier detection. Predictive performance between our joint models and the QCancer Colorectal model on the same patient cohort is yet to be explored, if feasible.

The one, non-static model is the ColonFlag (machine-learning algorithm) by Kinar 2016 [16]. Predictive performance for two-year risk in UK patients is similar between our joint models (c-statistic: ~0.75) and the ColonFlag model (c-statistic: 0.78), based on an existing external validation study [17]. This existing validation study also reported a ROC curve for 18–24-month risk from the ColonFlag model, to which the ROC curves presented here are very comparable. Our joint models use methodology that perform less parameter estimation than machine-learning, which requires much larger sample sizes and more computational capacity [22], and employs statistical methods, which are more accepted in healthcare, easier to understand, and therefore easier to embed into practice. It is also easier using our model to explain to a patient why we believe they are at risk of colorectal cancer, and therefore why further investigation and follow up are justified.

### 4.3. Implications for Practice

The joint models are designed to provide an up-to-date risk prediction when an FBC is added to the patient’s record. The most recent FBC was considered baseline, which mimics practice because the most recent FBC is considered when examining a patient and historical FBCs are often not considered unless the change is very obvious. More subtle trends (including changes within the reference ranges, unlikely to be noticed by a clinician) would be considered by our joint models. Additionally, the models are designed to use routinely available data and we envisage they would be programmed into practice software to run automatically when a new FBC becomes available. Therefore, there will likely be no additional work for patients or GP staff to identify a patient’s risk of diagnosis from our joint models.

Over the last 12 months, faecal immunochemical test (FIT) testing, which examines stool samples for traces of blood, has proved a useful test outside the screening programme for use in patients with low-risk symptoms. Negative findings on FIT avoids the need for colonoscopy, based on a 98% NPV in a recent primary care study [38]. However, these patients are nonetheless symptomatic, which is problematic for early detection because symptoms likely present in late-stage disease [5,37]. Our joint models use only data recorded earlier than two years prior to diagnosis, a time where patients are likely asymptomatic [5]. Patients identified as high-risk from our models could be referred for FIT testing, which is much more practical, cheaper and less invasive than colonoscopy, is an accurate test (85% sensitivity [39]), and carries less burden on the patient and the health system. However, the NPVs in asymptomatic patients identified from our joint models would need to be investigated further.

### 4.4. Strengths and Limitations

#### 4.4.1. Strengths

A large sample size and duration of follow-up was used to develop the joint models, which exceeded the sample size requirements. The joint models therefore had little-to-no overfitting or optimistic performance, with comparable performance between development and validation cohorts. Therefore, performance measures can be considered reliable.

Multivariate joint models were developed, which use repeated measures data. This is a key strength and improvement on existing prediction models, as joint models provide a more individualised risk assessment. The models use changes in three blood levels commonly available in primary care that have little-to-no correlation (analyses not reported here—available from the authors). 

One strength of the internal validation is that it used a large sample size to make an overall assessment of the joint models. This increases the precision of estimates and reliability of performance measures. Another strength is that predictive performance of these joint models were compared to the ColonFlag algorithm using the same patient cohort. This eliminates heterogeneity among patient samples to ensure a direct comparison of models. Many models for colorectal cancer detection exist, but no study has directly compared these to the ColonFlag algorithm, which is receiving much attention in UK general practices.

#### 4.4.2. Limitations

The joint models rely on routinely available FBCs to identify risk of diagnosis. Their impact is therefore limited to patients who are referred for blood testing by their primary care GP. However, colorectal cancer is more common in older age groups, where FBCs are also more commonly performed, so it is likely the model will have higher impact in the patient group most at risk of diagnosis. 

FBC blood tests are ordered for many reasons in primary care, not colorectal cancer specifically, but these reasons are not available in CPRD. It is possible that patients without colorectal cancer who have many FBCs in the five-year period have another disease or condition that influences blood levels over time. Therefore, some false positives (patients determined to be high risk who are not diagnosed with colorectal cancer) may have another illness. Data on comorbidities, including other cancers, will be obtained and considered as future work.

Existing systematic reviews have identified many risk factors for colorectal cancer, including alcohol consumption levels, ethnicity, and family history of colorectal cancer [40,41]. Not all risk factors are available or accurately recorded in CPRD or were available in our dataset so were not included in the models. However, we have provided detailed reasons for why age, sex, and FBC parameters were appropriate as the only covariates in the models at this stage. As future work, further risk factors may be obtained and considered as covariates.

Although a large sample and number of events were used for the overall assessment of the joint models in the internal validation, the sample size and number of events were small for some subgroups. Performance of the models in relevant subgroups should therefore be assessed in larger samples to increase precision.

Tumour staging was missing for approximately 40% of diagnoses. However, where known, there was a greater proportion of patients diagnosed with stage A cancer than stage D. The bulk of diagnosis were at stage B or C. Overall, there were more stage A + B tumours than stage C + D tumours, indicating more early-stage diagnoses than late stage in our dataset. Model performance for detecting early-stage tumours will be assessed in larger staging subgroups as future work.

## 5. Conclusions and Further Work

We developed a multivariate joint model for males and females separately. The models use routinely available FBC data earlier than two years before diagnosis to identify relevant trends that contribute to two-year risk of colorectal cancer. The models perform well and similarly to the ColonFlag algorithm, which is receiving much attention in clinical practice, but our joint models are more transparent and easier to interpret and embed into clinical practice. Performance of the joint models in relevant subgroups was also assessed but were limited to small sample sizes. Further work is therefore required. For example, the models require external validation using patients from primary care practices not involved in the development or internal validation process. 

We have planned an external validation study using CPRD AURUM data, consisting of practices that do not contribute to the CPRD GOLD database used in this study. This database is much larger than CPRD GOLD so would provide larger patient samples to assess performance in subgroups. Further subgroups, including tumour characteristics, such as early- vs. late-stage tumours, would be considered. External validation comparing several models for colorectal cancer detection in similar populations is planned. Additionally, we plan to perform decision curve analyses, which balance the benefits with drawbacks of intervention in clinical practice due to the prediction model [42,43,44,45,46,47,48], and assess performance of FIT screening in high-risk asymptomatic patients identified by our models. Future work also includes exploring the addition of further risk factors for colorectal cancer and expanding the models to include trends in other common types of blood tests and for various cancers (using trends specific to those cancers).

## Figures and Tables

**Table 1:** Summary of patient characteristics.

Summary Statistic	Males		Females	
	Diagnosed	Not Diagnosed	Diagnosed	Not Diagnosed
***Development cohort:***				
**No. (%)**	865 (0.4%)	249,851 (99.6%)	677 (0.3%)	246,018 (99.7%)
**Mean age ^1^ (SD)**	70.9 (10.0)	60.7 (13.0)	73.2 (11.0)	61.9 (14.6)
**Age ^1^ range**	40–95	40–104	40–96	40–108
***Internal validation cohort:***				
	**Males**		**Females**	
	**Diagnosed**	**Not diagnosed**	**Diagnosed**	**Not diagnosed**
**No. (%)**	1,040 (0.3%)	311,404 (99.7%)	1,200 (0.3%)	461,700 (99.7%)
**Mean age ^1^ (SD)**	71.6 (10.2)	60.6 (13.0)	73.4 (11.2)	61.7 (14.6)
**Age ^1^ range**	40–95	40–109	40–98	40–107

^1^ Age (years) at baseline FBC.

**Table 2:** Cox sub-model from the joint models.

Variable	Males	Females
	HR (95% CI)	HR (95% CI)
Age ^2^ (years) ^1^	1.015 (1.013, 1.017)	1.014 (1.012, 1.016)
Age ^2^ × log(Age) (years) ^1^	0.997 (0.997, 0.997)	0.997 (0.997, 0.998)
Trend: haemoglobin (g/dL) ^2^	0.868 (0.824, 0.916)	0.863 (0.805, 0.926)
Trend: mean cell volume (fL) ^2^	0.996 (0.983, 1.009)	0.986 (0.972, 1.000)
Trend: platelets (10^12^/L) ^2^	1.001 (0.999, 1.002)	1.002 (1.001, 1.003)
***Baseline two-year survival *** **^3^**	***0.999941***	***0.9999618***

^1^ Age (years) at baseline FBC (most recent FBC available prior to two years before diagnosis/censor). ^2^ These HRs indicate how an increase in the patient’s blood parameter from the average population trend effects risk of diagnosis. ^3^ Breslow estimate. Abbreviations: HR = hazard ratio; CI = confidence interval.

**Table 3:** Performance measures of the joint models.

Performance Measure	Males		Females	
	Development	Validation	Development	Validation
Brier score	0.0034	0.0033	0.0027	0.0028
R_D_^2^	0.28	0.30	0.31	0.34
C-statistic	0.739 (95% CI = 0.726–0.753)	0.751 (95% CI = 0.739–0.764)	0.753 (95% CI = 0.737–0.769)	0.763 (95% CI = 0.753–0.775)
D-statistic	1.27 (95% CI = 1.16–1.38)	1.33 (95% CI = 1.23–1.43)	1.38 (95% CI = 1.26–1.51)	1.46 (95% CI = 1.37–1.55)
Calibration slope	1.00	1.06	1.00	1.05

**Table 4:** Diagnostic accuracy measures of the joint models (validation cohort).

Risk Centile	Risk Cut-Off	True Positives	False Positives	True Negatives	False Negatives	Sensitivity (%)	Specificity (%)	PPV (%)	NPV (%)
***Males:***									
75%	0.3670%	600	77511	233893	440	57.69	75.11	0.77	99.81
80%	0.4036%	505	61984	249420	535	48.56	80.10	0.81	99.79
85%	0.4406%	401	46466	264938	639	38.56	85.08	0.86	99.76
90%	0.4839%	291	30954	280450	749	27.98	90.06	0.93	99.73
95%	0.5525%	180	15443	295961	860	17.31	95.04	1.15	99.71
99%	0.7232%	49	3076	308328	991	4.71	99.01	1.57	99.68
***Females:***									
75%	0.2767%	710	115018	346682	490	59.17	75.09	0.61	99.86
80%	0.3043%	614	91967	369733	586	51.17	80.08	0.66	99.84
85%	0.3348%	513	68922	392778	687	42.75	85.07	0.74	99.83
90%	0.3747%	397	45893	415807	803	33.08	90.06	0.86	99.81
95%	0.4426%	237	22909	438791	963	19.75	95.04	1.02	99.78
99%	0.6446%	75	4554	457146	1125	6.25	99.01	1.62	99.75

## Data Availability

The datasets used in this study are available from CPRD but restrictions apply [19].

## Supplementary Materials

The following supporting information can be downloaded at: https://www.mdpi.com/article/10.3390/cancers14194779/s1, File S1: Supplementary, Table S1: Summary of FBC data and follow-up (development cohort), Table S2: Summary of FBC data and follow-up (internal validation cohort), Table S3: Duke’s tumour stage (diagnosed patients only), Table S4: Mixed-effects sub-models from the joint models (fixed effects), Table S5: Mixed-effects sub-models from the joint models (random effects: variance-covariance matrix1), Table S6: Summary of FBC data and follow-up by age (at baseline) group (validation cohort), Table S7: Summary of patient characteristics, FBC data, and follow-up by number of FBCs (validation cohort), Table S8: Summary of patient characteristics and FBC data by time span on FBCs (validation cohort), Figure S1: C-statistic for the joint models and ColonFlag by age (at baseline) group in males (top) and females (bottom) (validation cohort), Figure S2: Calibration plots for the joint models by age (at baseline) group in males (left) and females (right) (validation cohort), Figure S3: C-statistic for the joint models and ColonFlag by number of FBCs in males (top), females (middle), and males aged 70–89 years at baseline FBC (bottom) (validation cohort), Figure S4: Calibration plots for the joint models by number of FBCs in males (top left) and females (top right) in the validation cohort and males in the development cohort (bottom left), Figure S5: C-statistic for the joint models and ColonFlag by time span of FBCs in males (top) and females (bottom) (validation cohort), Figure S6: Calibration plots for the joint models by time span of FBCs in males (left) and females (right) (validation cohort) [8,13,14,16,17,20,21,28,32,40,49,50,51,52,53,54,55,56,57,58,59,60,61,62,63,64,65,66,67,68,69].
