# Supplementary material for: Full Blood Count Trends for Colorectal Cancer Detection in Primary Care: Development and Validation of a Dynamic Prediction Model
Source: Cancers (Basel). 2022 Sep 29;14(19):4779. doi: 10.3390/cancers14194779 (PMC9563332; doi:10.3390/cancers14194779)
Supplement: Supplementary file 1 [file cancers-14-04779-s001.zip › cancers-1906816-supplementary.pdf]

Supplementary Methods

Study design

A hypothetical patient is described here to help clarify the study design. Assume the date is June 2022. A patient visits their GP, who orders a FBC blood test. This FBC was measured in June 2022 and has now entered the patient's electronic GP record. This current FBC is considered baseline and corresponds to the end of the five-year longitudinal period (June 2022, time=5). The start of the five-year longitudinal period is therefore five years earlier (June 2017, time=0). Assume the patient already had four FBCs in the past, with the earliest measured in September 2017. The earliest is then measured at time=0.25 years into this five-year period (i.e. three months after the start of the longitudinal period: June 2017 to September 2017). Trends over historic FBCs in this five-year period up to the current/baseline FBC (time=0 to 5) are used to identify risk of diagnosis two years in the future following baseline FBC.

Predictors

A list of reasons why only age, sex, and FBC results were considered:

1. A recent systematic review identified many predictors included in existing prediction models for bowel cancer[1]. Age and sex were the most common predictors and others included body mass index, ethnicity, family history, and various factors related to lifestyle, diet, and medication use. It is generally known that many of these additional predictors are not accurately recorded in EHRs, particularly ethnicity, family history, and alcohol consumption levels. Although relevant to the disease, using inaccurately recorded predictors could compromise reliability of the models.

2. The ColonFlag model, which is actively being used in Israel, relies on only the age, sex, and FBC results to score patients[2]. Discriminative ability of the model is reportedly good at 18-24 months prior to diagnosis (c-statistic = 0.78) in UK patient data[3]. Our hypothesis *a priori* was therefore that age, sex, and FBC results alone may be sufficient to identify risk of diagnosis accurately.

3. Almost anything can influence FBC results and be considered confounders[4-6]. These include medication use, such as aspirin[7], and co-morbidities, such as cardiovascular disease[8]. Some of these confounding variables are not available in EHR datasets. For example, diet and vitamin supplementation can effect haemoglobin-related parameters[9]]and exercise levels can effect haemoglobin-, platelet-, and white blood cell-related parameters[10]. Level of sleep is known to effect white blood cell-related parameters[11, 12]. Additionally, altitude is known to effect haemoglobin-related parameters, with high altitude hypoxia increasing blood levels[13-16].

4. Joint models are computationally intensive to develop. It may not have been possible to include many predictors in the model without compromising other data, such as reducing the sample size, to make the development practical and achievable.

A list of reasons why only haemoglobin, MCV, and platelets were used out of all FBC parameters:

1. *Clinical relevance*: iron-deficiency anaemia is a known risk factor for colorectal cancer used in practice to aid referral for further testing[17, 18]. It can be detected using haemoglobin and MCV results from a FBC (as per NICE and WHO national guidelines)[19, 20]. Using trends in these two parameters could capture patients en-route to developing iron-deficiency anaemia, i.e. before

national thresholds for microcytic anaemia, which may be due to iron-deficiency anaemia, are reached.

2. *Systematic review findings*: our recent systematic review identified six relevant parameters, of which haemoglobin, MCV, and platelets were either statistically significant in meta-analysis or consistently statistically significantly different between patients with and without colorectal cancer in each study that analysed the blood level[21].

3. *Amount of missing data*: our previous data quality assessment identified that these three parameters had the least amount of missing data among all 20 FBC parameters, missing in less than 7% of FBCs[22]. Using these commonly available parameters would maximise the number of FBCs used to identify trends.

4. *Correlation*: many parameters are mathematically related so would be highly correlated[22]. including correlated predictors in a single model can lead to bias in the estimation of regression coefficients[23, 24]. These three parameters are not mathematically related and have little-to-no correlation in levels and trends (analyses not reported here – available from the authors). These three parameters each reflect a different aspect of the blood.

5. *Computational intensiveness*: including many more parameters would increase computer capacity and time taken to build the model. Limiting to these three parameters makes the modelling practical and achievable.

Model development

Joint models: in the multivariate joint model setting, a mixed-effects sub-model is developed for each longitudinal outcome separately. Changes over time identified in the mixed-effects models contribute to a single Cox sub-model for risk prediction through additional parameters[25]. These parameters are referred to as association parameters, captured by a multivariate latent Gaussian process[26]. This process involves first extracting the best linear unbiased predictor (BLUP) of each random effect in the mixed-effects sub-model[24, 27]. BLUPs are estimates of the random effects for individual groups, depending on how the models were structured. For example, patients (i.e. the group) can be modelled using random effects to account for correlation/covariance between and within repeated measures in individual patients and the BLUPs are values that incorporate this information. The next step in the process is to take the linear combination of BLUPs for each longitudinal outcome separately and subsequently include them as time-varying covariates in the Cox model.

Choice of linear splines in mixed-effect sub-models: the choice of linear splines and knot locations was based on our previous work[28]. We determined linear splines as the better fitting approach based on visual inspection of LOWESS trends and Akaike and Bayesian information criteria, having compared piecewise linear splines, restricted cubic splines, and fractional polynomials, and varying number of knots and knot locations.

Choice of fractional polynomials in the Cox sub-model: the choice of fractional polynomials was because these were the better fitting approach based on Akaike and Bayesian information criteria and the c-statistic, having compared various functional forms: linear, categorised, linear splines, and restricted cubic splines. Additionally, fractional polynomials did not violate the proportional hazards assumption (assessed using plots of Schoenfeld residuals). Analyses not reported here – available from the authors.

Model control arguments and parameters: the multivariate joint model was developed using the *mjoint()* command in the *JoineRML* package in R software[29]. By default, the *JoineRML* package performs 100 burn-in iterations per longitudinal parameter during maximum likelihood estimation (to derive each estimate in the model). Upon assessing model convergence using convergence plots for each estimate, 75 burn-in iterations were used in total. This reduction from 300 to 75 iterations still achieved convergence, but also sped up computation time, as fewer iterations were being performed to estimate parameters. Additionally, antithetic sampling was used, which is a type of Monte Carlo variance reduction technique to speed up convergence. A simulation study showed this approach offers the quickest computation time with large sample sizes compared to ordinary Monte Carlo and Quasi-Monte Carlo methods[30]. The study also shows that antithetic sampling gives comparable coverage rates to other methods, which represent the proportion of simulations where the estimate resides within the corresponding confidence interval[31].

Risk predictions: two-year risk predictions were estimated using the *dynSurv()* command in the *JoineRML* package. This command identifies the patient-specific trend from the mixed-effects sub-model for each FBC parameter, extracts the best linear unbiased predictors (BLUPs) (i.e. random effects) from these models, and includes them in a Cox model to identify each patient's event-free probability based on their FBC trend. This event-free probability incorporates the baseline survival estimate by default. The risk of diagnosis is then one minus this event-free probability.

Model validation

A conventional working laptop offers either 8 or 16 GB RAM, which is a built-in memory slot that stores data whilst being processed. We used an advanced 'super-computer' to develop the model, which offered approximately 130 GB RAM. However, this was still insufficient for the entire patient sample and could facilitate approximately 250,000 patients only. Therefore, the final development sample consisted of around 226,000 randomly chosen males and females separately, which used as much of the available computer capacity as possible, whilst making modelling achievable.

References

[1] Usher-Smith, J.A., Walter, F.M., Emery, J.D., Win, A.K., and Griffin, S.J., Risk Prediction Models for Colorectal Cancer: A Systematic Review. *Cancer Prev Res (Phila)*, 2016. 9(1): p. 13-26.

[2] Kinar, Y., Kalkstein, N., Akiva, P., Levin, B., Half, E.E., Goldshtein, I., Chodick, G., and Shalev, V., Development and validation of a predictive model for detection of colorectal cancer in primary care by analysis of complete blood counts: a binational retrospective study. *J Am Med Inform Assoc*, 2016. 23(5): p. 879-90.

[3] Birks, J., Bankhead, C., Holt, T.A., Fuller, A., and Patnick, J., Evaluation of a prediction model for colorectal cancer: retrospective analysis of 2.5 million patient records. *Cancer Med*, 2017. 6(10): p. 2453-2460.

[4] Lab Tests Online. Full Blood Count (FBC). 2020 [Accessed 4 August 2021]; Available from: https://labtestsonline.org.uk/tests/full-blood-count-fbc.

[5] myDr. Full blood count (FBC). 2017 [Accessed 4 August 2021]; Available from: https://www.mydr.com.au/tests-investigations/full-blood-count-fbc/.

[6] MedlinePlus. Complete Blood Count (CBC). 2020 [Accessed 4 August 2021]; Available from: https://medlineplus.gov/lab-tests/complete-blood-count-cbc/.

[7] Gaskell, H., Derry, S., and Moore, R.A., Is there an association between low dose aspirin and anemia (without overt bleeding)? Narrative review. *BMC Geriatr*, 2010. 10: p. 71.

[8] Lassale, C., Curtis, A., Abete, I., van der Schouw, Y.T., Verschuren, W.M.M., Lu, Y., and Bueno-de-Mesquita, H.B.A., Elements of the complete blood count associated with cardiovascular disease incidence: Findings from the EPIC-NL cohort study. *Sci Rep*, 2018. 8(1): p. 3290.

[9] Better Health Channel. Blood count. 2019 [Accessed 4 August 2021]; Available from: https://www.betterhealth.vic.gov.au/health/conditionsandtreatments/blood-count.

[10] Arakawa, K., Hosono, A., Shibata, K., Ghadimi, R., Fuku, M., Goto, C., Imaeda, N., Tokudome, Y., Hoshino, H., Marumoto, M., et al., Changes in blood biochemical markers before, during, and after a 2-day ultramarathon. *Open Access J Sports Med*, 2016. 7: p. 43-50.

[11] Boudjeltia, K.Z., Faraut, B., Stenuit, P., Esposito, M.J., Dyzma, M., Brohee, D., Ducobu, J., Vanhaeverbeek, M., and Kerkhofs, M., Sleep restriction increases white blood cells, mainly neutrophil count, in young healthy men: a pilot study. *Vasc Health Risk Manag*, 2008. 4(6): p. 1467-70.

[12] Liu, H., Wang, G., Luan, G., and Liu, Q., Effects of sleep and sleep deprivation on blood cell count and hemostasis parameters in healthy humans. *J Thromb Thrombolysis*, 2009. 28(1): p. 46-9.

[13] Bain, B.J., A Beginner’s Guide to Blood Cells. 2nd ed. 2004, Malden, Massachusetts, USA: Blackwell Pub.

[14] Bain, B.J., Blood Cells: A Practical Guide. 5th ed. 2015, The Atrium, Southern Gate, Chichester, West Sussex, UK: John Wiley & Sons Ltd.

[15] Akunov, A., Sydykov, A., Toktash, T., Doolotova, A., and Sarybaev, A., Hemoglobin Changes After Long-Term Intermittent Work at High Altitude. *Front Physiol*, 2018. 9: p. 1552.

[16] Rowles, P.M. and Williams, E.S., Abnormal red cell morphology in venous blood of men climbing at high altitude. *Br Med J (Clin Res Ed)*, 1983. 286(6375): p. 1396.

[17] National Institute for Health and Care Excellence. Suspected cancer recognition and referral. 2020 [Accessed 20 October 2021]; Available from: https://www.nice.org.uk/guidance/ng12/resources/suspected-cancer-recognition-and-referral-pdf-1837268071621.

[18] Word Health Organisation. Guide to cancer early diagnosis. 2020 [Accessed 5 August 2021]; Available from: https://www.who.int/cancer/publications/cancer_early_diagnosis/en/.

[19] National Institute for Health and Care Excellence. What investigations should I arrange to confirm iron deficiency anaemia? 2021 [Accessed 20 October 2021]; Available from: https://cks.nice.org.uk/topics/anaemia-iron-deficiency/diagnosis/investigations/.

[20] World Health Organisation. Haemoglobin concentrations for the diagnosis of anaemia and assessment of severity. 2011 [Accessed 27 January 2021]; Available from: https://apps.who.int/iris/bitstream/handle/10665/85839/WHO_NMH_NHD_MNM_11.1_eng.pdf?ua=1.

[21] Virdee, P.S., Marian, I.R., Mansouri, A., Elhussein, L., Kirtley, S., Holt, T., and Birks, J., The Full Blood Count Blood Test for Colorectal Cancer Detection: A Systematic Review, Meta-Analysis, and Critical Appraisal. *Cancers (Basel)*, 2020. 12(9).

[22] Virdee, P.S., Fuller, A., Jacobs, M., Holt, T., and Birks, J., Assessing data quality from the Clinical Practice Research Datalink: a methodological approach applied to the full blood count blood test. *Journal of Big Data*, 2020. 7(96): p. 1-18.

[23] Steyerberg, E.W., Clinical Prediction Models: A Practical Approach to Development, Validation, and Updating. 1st ed. 2009, New York, USA: Springer.

[24] Fitzmaurice, G.M., Laird, N.M., and Ware, J.H., Applied Longitudinal Analysis. 2nd ed. 2011: John Wiley & Sons.

[25] Asar, O., Ritchie, J., Kalra, P.A., and Diggle, P.J., Joint modelling of repeated measurement and time-to-event data: an introductory tutorial. Int J Epidemiol, 2015. 44(1): p. 334-44.

[26] Roberts, S., Osborne, M., Ebden, M., Reece, S., Gibson, N., and Aigrain, S., Gaussian processes for time-series modelling. Philos Trans A Math Phys Eng Sci, 2013. 371(1984): p. 20110550.

[27] Liu, X., Rong, J., and Liu, X., Best linear unbiased prediction for linear combinationsin general mixed linear models. Journal of Multivariate Analysis 2008. 99(8): p. 1503–1517.

[28] Virdee, P.S., Patnick, P., Watkinson, P., Birks, J., Holt, T. Trends in the full blood count blood test and colorectal cancer detection: a longitudinal, case-control study of UK primary care patient data. *NIHR Open Research*, 2022, 2, 32:1-53. DOI: 10.3310/nihropenres.13266.1.

[29] Hickey, G.L., Philipson, P., Jorgensen, A., and Kolamunnage-Dona, R., joineRML: a joint model and software package for time-to-event and multivariate longitudinal outcomes. *BMC Med Res Methodol*, 2018. 18(1): p. 50.

[30] Philipson, P., Hickey, G.L., Crowther, M.J., and Kolamunnage-Dona, R., Faster Monte Carlo estimation of joint models for time-to-event and multivariate longitudinal data. *Computational Statistics and Data Analysis*, 2020. 151: p. 1-14.

[31] Benoit, J.S., Chan, W., and Doody, R.S., Joint coverage probability in a simulation study on Continuous-Time Markov Chain parameter estimation. *J Appl Stat*, 2015. 42(12): p. 2531-2538.

Supplementary Results

Summary of patient data

Table S1: Summary of FBC data and follow-up (development cohort)

	Males		Females	
	Diagnosed	Not diagnosed	Diagnosed	Not diagnosed
**No. FBCs in total**	3,774	801,390	3,573	909,569
**Mean no. FBCs (SD)**	5.6 (7.8)	4.4 (6.8)	7.0 (9.0)	5.3 (8.1)
Age 40-49 years[1]	*2.6 (1.7)*	*2.9 (4.8)*	*2.8 (1.8)*	*3.5 (5.5)*
Age 50-59 years[1]	*5.7 (8.0)*	*4.0 (7.6)*	*6.5 (9.3)*	*4.9 (7.9)*
Age 60-69 years[1]	*4.1 (4.9)*	*4.6 (7.1)*	*10.8 (13.1)*	*5.8 (9.2)*
Age 70-79 years[1]	*5.9 (8.4)*	*5.2 (7.2)*	*5.9 (7.3)*	*6.4 (9.3)*
Age 80-89 years[1]	*6.9 (9.1)*	*5.0 (5.5)*	*5.7 (5.8)*	*5.6 (7.4)*
Age 90+ years[1]	*5.0 (4.5)*	*4.3 (4.1)*	*3.8 (2.3)*	*4.6 (5.5)*
**Median no. FBCs (range)**	3 (1-61)	2 (1-193)	3 (1-70)	3 (1-172)
Age 40-49 years[1]	*2 (1-8)*	*2 (1-75)*	*2 (1-8)*	*2 (1-86)*
Age 50-59 years[1]	*3 (1-41)*	*2 (1-193)*	*3 (1-41)*	*2 (1-122)*
Age 60-69 years[1]	*3 (1-39)*	*3 (1-82)*	*5 (1-70)*	*3 (1-172)*
Age 70-79 years[1]	*3 (1-59)*	*3 (1-105)*	*4 (1-44)*	*3 (1-149)*
Age 80-89 years[1]	*4 (1-61)*	*3 (1-85)*	*4 (1-35)*	*3 (1-90)*
Age 90+ years[1]	*4 (1-18)*	*3 (1-51)*	*3 (1-10)*	*3 (1-70)*
**Mean (SD) Hb (g/dL)**	13.7 (1.8)	14.3 (1.6)	12.7 (1.5)	13.0 (1.4)
Patients with 1 test	14.1 (1.6)	14.7 (1.4)	13.0 (1.5)	13.2 (1.3)
Patients with 2 tests	14.0 (1.7)	14.5 (1.5)	12.9 (1.4)	13.2 (1.3)
Patients with 3 tests	13.9 (1.7)	14.4 (1.5)	12.9 (1.4)	13.1 (1.4)
Patients with 4 tests	13.7 (1.8)	14.2 (1.6)	12.9 (1.4)	13.0 (1.4)
Patients with 5 tests	13.7 (1.7)	14.0 (1.6)	12.7 (1.5)	12.9 (1.4)
Patients with 6 tests	13.5 (1.9)	13.9 (1.7)	12.7 (1.4)	12.8 (1.4)
Patients with 7 tests	13.3 (1.8)	13.7 (1.7)	12.5 (1.6)	12.7 (1.5)
Patients with 8 tests	13.2 (2.0)	13.5 (1.8)	12.3 (1.7)	12.6 (1.5)
Patients with 9 tests	12.9 (2.1)	13.4 (1.8)	12.2 (1.5)	12.5 (1.5)
Patients with 10 tests	12.8 (2.2)	13.3 (1.8)	12.1 (1.5)	12.4 (1.5)
Patients with >10 tests	12.7 (1.8)	13.3 (1.7)	12.4 (1.4)	12.4 (1.4)
**Mean (SD) MCV (fL)**	91.8 (6.6)	91.3 (5.6)	90.6 (6.6)	90.5 (6.1)
Patients with 1 test	91.6 (5.8)	91.2 (5.2)	90.4 (6.3)	90.5 (5.7)
Patients with 2 tests	91.5 (6.1)	91.2 (5.4)	90.2 (6.2)	90.4 (5.8)
Patients with 3 tests	91.4 (6.3)	91.3 (5.6)	90.3 (6.2)	90.3 (6.0)
Patients with 4 tests	91.4 (6.2)	91.3 (5.7)	90.3 (6.6)	90.3 (6.1)
Patients with 5 tests	91.2 (5.8)	91.4 (5.8)	90.4 (6.2)	90.2 (6.3)
Patients with 6 tests	91.9 (6.5)	91.4 (6.1)	90.3 (6.5)	90.2 (6.4)
Patients with 7 tests	92.2 (7.2)	91.5 (6.2)	90.4 (6.5)	90.3 (6.5)
Patients with 8 tests	91.0 (6.8)	91.5 (6.4)	90.3 (7.2)	90.3 (6.7)
Patients with 9 tests	90.8 (8.3)	91.5 (6.4)	90.7 (7.7)	90.5 (6.7)
Patients with 10 tests	91.6 (8.2)	91.6 (6.4)	91.0 (7.5)	90.5 (6.8)
Patients with >10 tests	94.2 (8.2)	92.4 (6.5)	91.7 (6.0)	91.9 (6.7)

**Mean (SD) platelets (10^9/L)**	242.2 (77.6)	247.3 (76.1)	294.6 (85.3)	282.4 (81.7)
Patients with 1 test	244.8 (74.8)	247.5 (69.5)	287.2 (84.3)	279.0 (74.8)
Patients with 2 tests	240.1 (74.5)	246.6 (72.6)	288.4 (85.6)	280.0 (77.3)
Patients with 3 tests	244.5 (83.9)	245.7 (74.5)	288.6 (83.1)	280.7 (78.9)
Patients with 4 tests	244.4 (79.6)	244.9 (76.6)	287.1 (82.3)	282.1 (81.8)
Patients with 5 tests	238.5 (72.9)	244.9 (79.3)	297.3 (85.0)	282.8 (83.8)
Patients with 6 tests	242.5 (75.5)	245.2 (81.9)	295.2 (91.1)	284.5 (86.9)
Patients with 7 tests	235.1 (82.1)	245.9 (85.2)	302.2 (85.4)	285.8 (90.4)
Patients with 8 tests	229.8 (72.4)	246.7 (86.4)	302.5 (88.7)	287.4 (92.4)
Patients with 9 tests	242.4 (101.0)	247.8 (88.5)	307.4 (99.0)	288.3 (92.2)
Patients with 10 tests	246.2 (83.2)	248.3 (89.4)	294.4 (89.1)	289.4 (93.5)
Patients with >10 tests	242.4 (76.4)	257.4 (92.7)	310.8 (82.1)	293.3 (95.4)
**Median (range) Hb (g/dL)**	13.9 (5.4-19.7)	14.5 (0.4-21.0)	12.9 (5.3-17.1)	13.1 (0.4-21.0)
Patients with 1 test	14.3 (6.2-18.3)	14.8 (1.5-21.0)	13.1 (6.8-17.1)	13.3 (1.5-21.0)
Patients with 2 tests	14.2 (5.4-19.7)	14.7 (2.2-20.9)	13.1 (6.9-16.2)	13.3 (0.4-20.9)
Patients with 3 tests	14.1 (6.2-17.8)	14.5 (2.8-20.8)	13.0 (6.8-16.8)	13.2 (1.5-20.6)
Patients with 4 tests	13.9 (6.3-18.5)	14.4 (0.4-20.9)	13.0 (5.7-16.5)	13.1 (1.5-20.6)
Patients with 5 tests	13.8 (8.5-17.2)	14.2 (1.2-20.9)	12.9 (7.6-16.3)	13.0 (2.5-20.9)
Patients with 6 tests	13.7 (8.0-17.2)	14.1 (4.4-20.4)	12.8 (8.7-15.8)	12.9 (2.5-19.7)
Patients with 7 tests	13.2 (7.8-17.1)	13.9 (3.9-20.3)	12.8 (7.2-16.2)	12.8 (3.8-20.7)
Patients with 8 tests	13.0 (8.7-18.7)	13.7 (4.6-21.0)	12.5 (8.3-15.6)	12.7 (2.6-20.7)
Patients with 9 tests	13.1 (6.8-17.7)	13.6 (5.3-20.9)	12.4 (7.5-15.4)	12.6 (4.5-20.1)
Patients with 10 tests	12.9 (7.5-18.3)	13.4 (4.6-20.0)	12.0 (8.7-15.2)	12.5 (4.1-20.8)
Patients with >10 tests	12.8 (5.7-17.8)	13.5 (1.0-21.0)	12.5 (5.3-16.2)	12.5 (1.5-20.0)
**Median (range) MCV (fL)**	92.0 (56.0-120.6)	91.2 (53.0-125.0)	90.7 (59.5-120.1)	90.8 (53.0-125.0)
Patients with 1 test	91.8 (65.9-120.6)	91.0 (53.0-125.0)	90.6 (60.2-114.9)	90.8 (53.6-125.0)
Patients with 2 tests	91.9 (56.0-113.3)	91.0 (53.3-125.0)	90.6 (64.2-117.0)	90.7 (53.0-125.0)
Patients with 3 tests	91.8 (59.3-119.9)	91.1 (53.0-125.0)	90.4 (63.0-112.3)	90.6 (53.1-125.0)
Patients with 4 tests	91.7 (66.0-114.8)	91.2 (54.7-124.7)	90.2 (62.1-116.6)	90.5 (53.4-125.0)
Patients with 5 tests	91.0 (67.0-116.2)	91.3 (54.3-124.3)	90.8 (59.5-109.3)	90.4 (54.3-124.6)
Patients with 6 tests	91.6 (67.0-115.9)	91.4 (55.7-123.2)	90.7 (73.6-113.3)	90.5 (53.1-124.7)
Patients with 7 tests	92.0 (66.0-117.9)	91.5 (57.8-124.6)	90.0 (67.0-118.2)	90.5 (55.4-125.0)
Patients with 8 tests	91.7 (58.5-107.5)	91.6 (56.8-124.3)	90.5 (70.8-120.1)	90.6 (54.5-125.0)
Patients with 9 tests	91.6 (64.1-115.5)	91.7 (56.6-124.0)	90.6 (72.2-116.3)	90.8 (53.4-125.0)
Patients with 10 tests	93.0 (61.4-105.8)	91.7 (57.0-123.0)	90.8 (68.0-113.8)	90.9 (54.7-125.0)
Patients with >10 tests	95.0 (58.2-119.7)	92.4 (57.0-125.0)	91.0 (70.0-108.2)	92.0 (57.8-125.0)
**Median (range) platelets (10^9/L)**	234 (0-873)	238 (0-1499)	282 (0-819)	273 (0-1469)
Patients with 1 test	234 (2-740)	239 (0-1410)	275 (95-603)	270 (1-1416)
Patients with 2 tests	230 (38-832)	238 (1-1489)	276 (0-800)	271 (0-1450)
Patients with 3 tests	235 (0-849)	237 (2-1410)	276 (70-696)	272 (0-1469)
Patients with 4 tests	237 (38-873)	235 (3-1369)	278 (50-703)	272 (0-1389)
Patients with 5 tests	234 (64-588)	235 (0-1303)	285 (105-596)	272 (0-1404)
Patients with 6 tests	233 (67-524)	235 (1-1431)	282 (102-819)	274 (0-1371)
Patients with 7 tests	233 (78-683)	235 (2-1499)	288 (112-546)	275 (3-1289)
Patients with 8 tests	233 (76-471)	236 (1-1027)	293 (111-619)	277 (4-1455)
Patients with 9 tests	226 (76-862)	237 (3-1054)	297 (111-629)	277 (3-1317)
Patients with 10 tests	231 (98-547)	237 (3-1387)	289 (94-594)	278 (6-1411)
Patients with >10 tests	239 (47-540)	246 (1-1452)	302 (98-530)	282 (2-1465)
**No. (%) missing Hb[2]**	25 (0.66%)	4,784 (0.60%)	34 (0.95%)	5,267 (0.58%)
**No. (%) missing MCV[2]**	174 (4.61%)	20,022 (2.50%)	182 (5.09%)	21,057 (2.32%)

**No. (%) missing platelets[2]**	150 (3.97%)	18,540 (2.13%)	167 (4.67%)	21,595 (2.37%)
**Median time (range)[3]**	2.2 (0-5.0)	1.5 (0-5.0)	2.7 (0-5.0)	2.0 (0-5.0)
**Median follow-up (range)[4]**	4.18 (1.75-7.18)	3.47 (1.75-7.25)	4.67 (1.76-7.21)	4.01 (1.75-7.25)

[1]Age at baseline FBC

[2]Percentages are out of total number of FBCs in that group.

[3]Time (years) between the first and last FBC in the five-year longitudinal period.

[4]Follow-up per patient is the time (years) from first FBC to diagnosis/censor.

Abbreviations: FBC=full blood count; Hb=haemoglobin; MCV=mean corpuscular volume.

Table S2: Summary of FBC data and follow-up (internal validation cohort)

	Males		Females	
	**Diagnosed**	**Not diagnosed**	**Diagnosed**	**Not diagnosed**
**No. FBCs in total**	4,513	1,002,342	6,370	1,704,897
**Mean no. FBCs (SD)**	5.3 (7.3)	4.6 (7.1)	8.6 (12.1)	5.3 (8.4)
**Median no. FBCs (range)**	3 (1-58)	2 (1-141)	4 (1-79)	3 (1-129)
**Mean (SD) Hb (g/dL)**	13.9 (1.7)	14.3 (1.6)	12.6 (1.5)	13.0 (1.4)
**Mean (SD) MCV (fL)**	90.4 (6.3)	91.3 (5.6)	90.3 (6.8)	90.5 (6.0)
**Mean (SD) platelets (10^9/L)**	248.3 (77.1)	246.1 (73.6)	289.9 (81.8)	281.5 (80.0)
**Median (range) Hb (g/dL)**	14.1 (5.8-19.1)	14.5 (0.4-21.0)	12.8 (4.5-17.6)	13.1 (0.4-21.0)
**Median (range) MCV (fL)**	91.0 (57.5-120.2)	91.1 (53.0-125.0)	90.6 (55.0-123.0)	90.7 (53.0-125.0)
**Median (range) platelets (10^9/L)**	236 (1-827)	237 (0-1,478)	280 (0-841)	272 (0-1,473)
**No. (%) missing Hb[1]**	33 (0.73%)	5,283 (0.53%)	51 (0.80%)	8,909 (0.52%)
**No. (%) missing MCV[1]**	104 (2.30%)	17,580 (1.75%)	209 (3.28%)	29,362 (1.72%)
**No. (%) missing platelets[1]**	119 (2.64%)	20,368 (2.03%)	229 (3.59%)	36,150 (2.12%)
**Median time (range)[2]**	2.3 (0-5.0)	1.5 (0-5.0)	2.5 (0-5.0)	2.0 (0-5.0)
**Median follow-up (range)[3]**	4.25 (1.75-7.19)	3.45 (1.75-7.25)	4.45 (1.76-7.19)	4.02 (1.75-7.25)

[1]Percentages are out of total number of FBCs in that group.
[2]Time (years) between the first and last FBC in the five-year longitudinal period.
[3]Follow-up per patient is the time (years) from first FBC to diagnosis/censor.
Abbreviations: FBC=full blood count; Hb=haemoglobin; MCV=mean corpuscular volume.

Table S3: Duke's tumour stage (diagnosed patients only)

Duke's stage	Males		Females	
	**Development (n=865)**	**Internal validation (n=1040)**	**Development (n=677)**	**Development (n=1200)**
**A**	78 (9.0%)	104 (10.0%)	73 (10.8%)	109 (9.1%)
**B**	180 (20.8%)	227 (21.8%)	128 (18.9%)	279 (23.3%)
**C**	188 (21.7%)	223 (21.4%)	147 (21.7%)	253 (21.1%)
**D**	71 (8.2%)	76 (7.3%)	37 (5.5%)	67 (5.6%)
**Unknown**	348 (40.2%)	410 (39.4%)	292 (43.1%)	492 (41.0%)

Model development

Table S4: Mixed-effects sub-models from the joint models (fixed effects)

Variable	Males (n=312,444, with 1,040 diagnosed)			Females (n=462,900, with 1,200 diagnosed)		
	**Haemoglobin (g/dL)**	**Mean corpuscular volume (fL)**	**Platelets (10^9/L)**	**Haemoglobin (g/dL)**	**Mean corpuscular volume (fL)**	**Platelets (10^9/L)**
Intercept	15.683 (95% CI= 15.514 - 15.851)	86.121 (95% CI= 85.848 - 86.394)	280.416 (95% CI= 277.644 - 283.189)	11.445 (95% CI= 11.318 - 11.572)	86.375 (95% CI= 86.100 - 86.65)	299.159 (95% CI= 296.281 - 302.038)
Age[1] (years)	-0.011 (95% CI= -0.014 - -0.008)	0.083 (95% CI= 0.078 - 0.089)	-0.400 (95% CI= -0.451 - -0.350)	0.036 (95% CI= 0.033 - 0.038)	0.068 (95% CI= 0.063 - 0.074)	-0.218 (95% CI= -0.271 - -0.164)
Age[1] – knot at 55 (years)		-0.042 (95% CI= -0.049 - -0.036)			-0.054 (95% CI= -0.061 - -0.047)	
Age[1] – knot at 60 (years)	-0.013 (95% CI= -0.020 - -0.007)		-0.140 (95% CI= -0.215 - -0.065)	-0.038 (95% CI= -0.044 - -0.033)		0.070 (95% CI= -0.008 - 0.148)
Age[1] – knot at 70 (years)	-0.026 (95% CI= -0.033 - -0.020)			-0.029 (95% CI= -0.036 - -0.023)		
Age[1] – knot at 80 (years)	-0.002 (95% CI= -0.010 - 0.005)			0.004 (95% CI= -0.002 - 0.009)		
Time[2] (years)	-0.039 (95% CI= -0.095 - 0.018)	0.032 (95% CI= 0.022 - 0.042)	-1.870 (95% CI= -2.044 - -1.696)	0.063 (95% CI= 0.021 - 0.105)	0.053 (95% CI= 0.042 - 0.063)	-1.417 (95% CI= -1.578 - -1.257)
Time[2] – knot at 3 (years)	0.128 (95% CI= 0.052 - 0.205)	0.166 (95% CI= 0.153 - 0.178)	-0.467 (95% CI= -0.721 - -0.214)	0.203 (95% CI= 0.143 - 0.262)	0.197 (95% CI= 0.185 - 0.21)	-1.278 (95% CI= -1.506 - -1.049)
Time[2] by age[1] interaction	0.001 (95% CI= -0.001 - 0.001)			-0.001 (95% CI= -0.002 - -0.001)		

Time[2] (knot at 3) by age[1] interaction	-0.001 (95% CI= -0.003 – 0.001)			-0.003 (95% CI= -0.004 - -0.002)		
Time[2] by age[1] (knot at 60) interaction	-0.004 (95% CI= -0.006 - -0.003)			-0.004 (95% CI= -0.006 - -0.003)		
Time[2] (knot at 3) by age[1] (knot at 60) interaction	0.005 (95% CI= 0.002 - 0.008)			0.007 (95% CI= 0.005 - 0.010)		
Time[2] by age[1] (knot at 70) interaction	0.003 (95% CI= 0.001 - 0.005)			0.004 (95% CI= 0.003 - 0.006)		
Time[2] (knot at 3) by age[1] (knot at 70) interaction	-0.009 (95% CI= -0.012 - -0.006)			-0.007 (95% CI= -0.010 - -0.004)		
Time[2] by age[1] (knot at 80) interaction	-0.005 (95% CI= -0.007 - -0.002)			-0.002 (95% CI= -0.003 – 0.001)		
Time[2] (knot at 3) by age[1] (knot at 80) interaction	0.008 (95% CI= 0.005 - 0.011)			0.003 (95% CI= 0.001 - 0.005)		

[1]Age (years) at baseline FBC (most recent FBC available prior to two years before diagnosis/censor).

[2]Time (years) from start of the five-year longitudinal period to the baseline FBC. The baseline FBC corresponds to the end of the five-year longitudinal period.

Table S5: Mixed-effects sub-models from the joint models (random effects: variance-covariance matrix[1])

			Haemoglobin (g/dL)		Mean corpuscular volume (fL)		Platelets (10^9/L)	
			Intercept[2]	Slope[2]	Intercept[2]	Slope[2]	Intercept[2]	Slope[2]
**Males**	**Haemoglobin (g/dL)**	**Intercept[2]**	1.62690	-0.148400	2.12050	-0.33343	-24.0980	4.2184
		**Slope[2]**		0.045538	-0.34763	0.08893	3.5333	-1.0635
	**Mean corpuscular volume (fL)**	**Intercept[2]**			26.77600	-1.83580	-58.4380	11.8150
		**Slope[2]**				0.58100	11.4880	-3.3446
	**Platelets (10^9/L)**	**Intercept[2]**					4171.0000	-248.1500
		**Slope[2]**						72.7390
	Residual variance		*0.7571097*		*2.3433855*		*39.8502589*	
**Females**	**Haemoglobin (g/dL)**	**Intercept[2]**	1.49890	-0.143890	3.65570	-0.50404	-24.5970	4.4482
		**Slope[2]**		0.042218	-0.52485	0.13220	3.8022	-1.1559
	**Mean corpuscular volume (fL)**	**Intercept[2]**			34.36400	-2.77550	-102.0600	18.3230
		**Slope[2]**				0.78968	17.1590	-4.9272
	**Platelets (10^9/L)**	**Intercept[2]**					5074.8000	-325.0500
		**Slope[2]**						96.4340
	Residual variance		*0.7295342*		*2.6443437*		*41.2218342*	

[1]Diagonals represent variance and non-diagonals represent co-variance

[2]Random intercept for patient and random slope for time over the five-year longitudinal period prior to the baseline FBC

Model performance in subgroups (internal validation cohort)

Table S6: Summary of FBC data and follow-up by age (at baseline) group (validation cohort)

	Males		Females	
	**Diagnosed (1,040)**	**Not diagnosed (311,404)**	**Diagnosed (1,200)**	**Not diagnosed (461,700)**
**No. (%) patients per group**				
Age 40-49 years	*22 (0.1%)*	*74,097 (99.9%)*	*39 (0.1%)*	*115,994 (99.9%)*
Age 50-59 years	*115 (0.2%)*	*74,631 (99.8%)*	*104 (0.1%)*	*99,093 (99.9%)*
Age 60-69 years	*257 (0.4%)*	*66,987 (99.6%)*	*239 (0.3%)*	*333,046 (99.7%)*
Age 70-79 years	*401 (0.9%)*	*42,676 (99.1%)*	*425 (0.6%)*	*65,852 (99.4%)*
Age 80-89 years	*224 (1.0%)*	*22,182 (99.0%)*	*331 (0.6%)*	*50,848 (99.4%)*
Age 90+ years	*21 (0.7%)*	*2,933 (99.3%)*	*62 (0.5%)*	*11,917 (99.5%)*
**No. FBCs in total**				
Age 40-49 years	*106*	*146171*	*98*	*295924*
Age 50-59 years	*388*	*207186*	*334*	*331387*
Age 60-69 years	*1086*	*239377*	*1270*	*333046*
Age 70-79 years	*1759*	*190923*	*2617*	*317750*
Age 80-89 years	*1056*	*109790*	*1756*	*253864*
Age 90+ years	*118*	*13914*	*295*	*52279*
**Median no. FBCs (range)**				
Age 40-49 years	*4 (1-21)*	*1 (1-141)*	*2 (1-8)*	*2 (1-134)*
Age 50-59 years	*2 (1-18)*	*2 (1-131)*	*2 (1-20)*	*2 (1-119)*
Age 60-69 years	*3 (1-58)*	*3 (1-123)*	*5 (1-70)*	*4 (1-189)*
Age 70-79 years	*3 (1-58)*	*3 (1-110)*	*4 (1-53)*	*4 (1-219)*
Age 80-89 years	*3 (1-48)*	*3 (1-84)*	*3 (1-52)*	*4 (1-144)*
Age 90+ years	*4 (1-22)*	*3(1-78)*	*4 (1-31)*	*3 (1-87)*
**No. (%) missing Hb[1]**				
Age 40-49 years	*1 (0.94%)*	*577 (0.39%)*	*2 (2.04%)*	*1186 (0.40%)*
Age 50-59 years	*2 (0.52%)*	*965 (0.47%)*	*1 (0.30%)*	*1483 (0.45%)*
Age 60-69 years	*8 (0.74%)*	*1152 (0.48%)*	*17 (1.34%)*	*1532 (0.46%)*
Age 70-79 years	*14 (0.80%)*	*976 (0.51%)*	*19 (0.73%)*	*1604 (0.50%)*
Age 80-89 years	*7 (0.66%)*	*687 (0.63%)*	*12 (0.68%)*	*1593 (0.63%)*
Age 90+ years	*1 (0.85%)*	*94 (0.68%)*	*0 (0.00%)*	*390 (0.75%)*
**No. (%) missing MCV[1]**				
Age 40-49 years	*2 (1.89%)*	*1767 (1.21%)*	*1 (1.02%)*	*4012 (1.36%)*
Age 50-59 years	*1 (0.26%)*	*3028 (1.46%)*	*8 (2.40%)*	*5020 (1.51%)*
Age 60-69 years	*26 (2.39%)*	*3798 (1.59%)*	*31 (2.44%)*	*4739 (1.42%)*
Age 70-79 years	*35 (1.99%)*	*3696 (1.94%)*	*120 (4.59%)*	*5948 (1.87%)*
Age 80-89 years	*28 (2.65%)*	*2681 (2.44%)*	*45 (2.56%)*	*5563 (2.19%)*
Age 90+ years	*12 (10.17%)*	*301 (2.16%)*	*4 (1.36%)*	*1073 (2.05%)*
**No. (%) missing platelets[1]**				
Age 40-49 years	*2 (1.89%)*	*2026 (1.39%)*	*3 (3.06%)*	*5588 (1.89%)*
Age 50-59 years	*10 (2.58%)*	*3713 (1.79%)*	*15 (4.49%)*	*6352 (1.92%)*
Age 60-69 years	*27 (2.49%)*	*4446 (1.86%)*	*51 (4.02%)*	*5921 (1.78%)*
Age 70-79 years	*37 (2.10%)*	*4135 (2.17%)*	*105 (4.01%)*	*6896 (2.17%)*
Age 80-89 years	*32 (3.03%)*	*2953 (2.69%)*	*52 (2.96%)*	*6430 (2.53%)*
Age 90+ years	*11 (9.32%)*	*349 (2.51%)*	*3 (1.02%)*	*1404 (2.69%)*

**Median time (range)[2]**				
Age 40-49 years	*1.0 (0-4.6)*	*0 (0-5.0)*	*0.8 (0-4.8)*	*0.5 (0-5.0)*
Age 50-59 years	*1.5 (0-4.9)*	*1.2 (0-5.0)*	*1.0 (0-4.9)*	*2.1 (0-5.0)*
Age 60-69 years	*2.5 (0-5.0)*	*2.3 (0-5.0)*	*2.0 (0-5.0)*	*2.6 (0-5.0)*
Age 70-79 years	*2.4 (0-5.0)*	*3.0 (0-5.0)*	*2.7 (0-5.0)*	*3.1 (0-5.0)*
Age 80-89 years	*2.4 (0-5.0)*	*3.1 (0-5.0)*	*2.8 (0-5.0)*	*3.1 (0-5.0)*
Age 90+ years	*2.9 (0-5.0)*	*2.8 (0-5.0)*	*2.4 (0-5.0)*	*2.6 (0-5.0)*
**Median follow-up (range)[3]**				
Age 40-49 years	*3.2 (1.8-6.5)*	*2.0 (1.8-7.2)*	*2.8 (1.9-6.8)*	*2.5 (1.8-7.2)*
Age 50-59 years	*3.5 (1.8-6.8)*	*3.2 (1.8-7.2)*	*3.1 (1.8-7.0)*	*4.1 (1.8-7.2)*
Age 60-69 years	*4.4 (1.8-7.2)*	*4.3 (1.8-7.2)*	*3.8 (1.8-7.1)*	*4.6 (1.8-7.2)*
Age 70-79 years	*4.3 (1.8-7.1)*	*5.0 (1.8-7.2)*	*4.7 (1.8-7.2)*	*5.1 (1.8-7.2)*
Age 80-89 years	*4.5 (1.8-7.2)*	*5.0 (1.8-7.2)*	*4.7 (1.8-7.0)*	*5.1 (1.8-7.2)*
Age 90+ years	*4.8 (1.8-6.9)*	*4.7 (1.8-7.2)*	*4.3 (1.8-7.0)*	*4.6 (1.8-7.2)*

[1]Percentages are out of total number of FBCs in that age group.
[2]Time (years) between the first and last FBC in the five-year longitudinal period.
[3]Follow-up per patient is the time (years) from first FBC to diagnosis/censor.
Abbreviations: FBC=full blood count; Hb=haemoglobin; MCV=mean corpuscular volume.

Figure S1: C-statistic for the joint models and ColonFlag by age (at baseline) group in males (top) and females (bottom) (validation cohort)

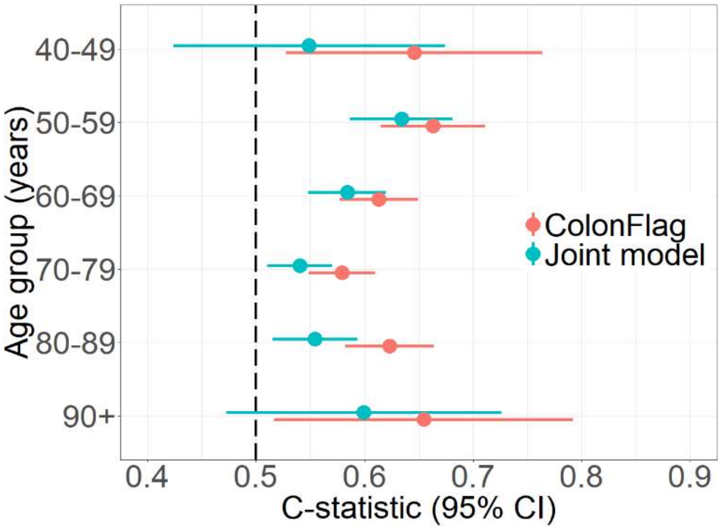

Number	Cases	Model	C-statistic (95%)
74119	22	●	0.549 (0.424, 0.674)
		●	0.646 (0.528, 0.764)
74746	115	●	0.634 (0.587, 0.682)
		●	0.663 (0.615, 0.711)
67244	257	●	0.584 (0.548, 0.620)
		●	0.613 (0.577, 0.649)
43077	401	●	0.540 (0.510, 0.570)
		●	0.579 (0.549, 0.610)
22406	224	●	0.554 (0.515, 0.593)
		●	0.623 (0.582, 0.664)
2954	21	●	0.599 (0.473, 0.726)
		●	0.655 (0.517, 0.792)

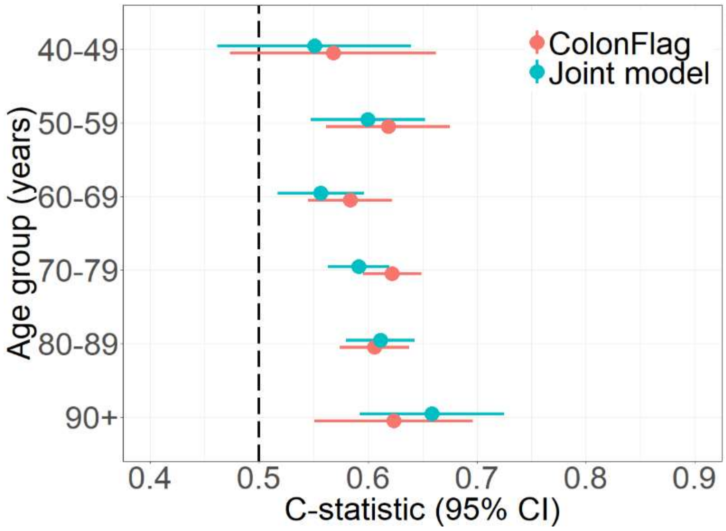

Number	Cases	Model	C-statistic (95%)
116033	39	●	0.551 (0.462, 0.640)
		●	0.568 (0.473, 0.662)
99197	104	●	0.600 (0.547, 0.652)
		●	0.618 (0.561, 0.675)
84283	239	●	0.557 (0.517, 0.596)
		●	0.583 (0.545, 0.622)
66277	425	●	0.591 (0.563, 0.620)
		●	0.622 (0.595, 0.649)
51179	331	●	0.611 (0.580, 0.643)
		●	0.606 (0.574, 0.638)
11979	62	●	0.659 (0.592, 0.725)
		●	0.624 (0.551, 0.696)

Figure S2: Calibration plots for the joint models by age (at baseline) group in males (left) and females (right) (validation cohort)

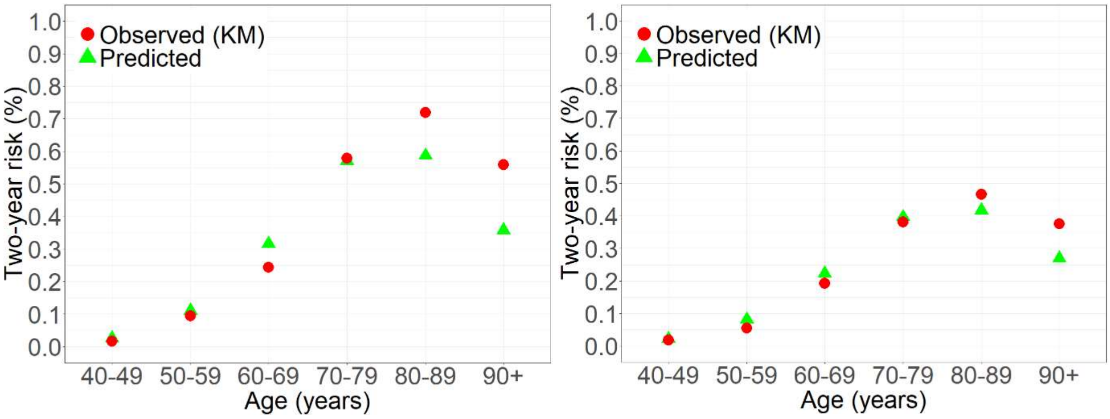

Abbreviations: KM=Kaplan-Meier

Table S7: Summary of patient characteristics, FBC data, and follow-up by number of FBCs (validation cohort)[1]

	Males		Females	
	Diagnosed (1,040)	Not diagnosed (311,404)	Diagnosed (1,200)	Not diagnosed (461,700)
**No. (%) patients per group**				
2	*189 (0.3%)*	*61,011 (99.7%)*	*190 (0.2%)*	*89,995 (99.8%)*
4	*127 (0.5%)*	*25,300 (99.5%)*	*129 (0.3%)*	*44,852 (99.7%)*
6	*64 (0.5%)*	*11,907 (99.5%)*	*82 (0.4%)*	*22,033 (99.6%)*
8	*38 (0.8%)*	*5,061 (99.3%)*	*39 (0.4%)*	*9,791 (99.6%)*
10	*26 (1.0%)*	*2,465 (99.0%)*	*21 (0.5%)*	*4,655 (99.5%)*
12	*13 (1.1%)*	*1,203 (98.9%)*	*12 (0.5%)*	*2,239 (99.5%)*
14	*4 (0.6%)*	*665 (99.4%)*	*4 (0.3%)*	*1,248 (99.7%)*
**Mean age (SD)[2]**				
2	*70.0 (10.0)*	*58.6 (12.0)*	*71.6 (12.6)*	*59.3 (13.9)*
4	*73.9 (9.8)*	*63.7 (12.1)*	*74.7 (10.0)*	*63.5 (14.1)*
6	*72.6 (8.1)*	*67.3 (11.8)*	*76.4 (11.9)*	*66.9 (13.9)*
8	*72.7 (10.8)*	*69.1 (11.6)*	*76.3 (9.3)*	*68.7 (13.9)*
10	*72.9 (7.7)*	*70.4 (11.7)*	*77.6 (8.8)*	*70.3 (13.7)*
12	*77.0 (8.9)*	*70.7 (12.2)*	*76.5 (10.2)*	*70.9 (13.6)*
14	*82.3 (4.4)*	*71.5 (12.0)*	*75.0 (2.9)*	*70.9 (13.5)*
**No. FBCs in total**				
2	*378*	*122022*	*380*	*179990*
4	*508*	*101200*	*516*	*179408*
6	*384*	*71442*	*492*	*132198*
8	*304*	*40488*	*312*	*78328*
10	*260*	*24650*	*210*	*46550*
12	*156*	*14436*	*144*	*26868*
14	*56*	*9310*	*56*	*17472*
**No. (%) missing Hb[3]**				
2	*1 (0.26%)*	*479 (0.39%)*	*3 (0.79%)*	*724 (0.40%)*
4	*4 (0.79%)*	*559 (0.55%)*	*12 (2.33%)*	*909 (0.51%)*
6	*6 (1.56%)*	*453 (0.63%)*	*1 (0.20%)*	*657 (0.50%)*
8	*0 (0.00%)*	*215 (0.53%)*	*0 (0.00%)*	*423 (0.54%)*
10	*2 (0.77%)*	*137 (0.56%)*	*1 (0.48%)*	*314 (0.67%)*
12	*0 (0.00%)*	*137 (0.95%)*	*0 (0.00%)*	*180 (0.67%)*
14	*0 (0.00%)*	*60 (0.64%)*	*0 (0.00%)*	*78 (0.45%)*
**No. (%) missing MCV[3]**				
2	*7 (1.85%)*	*1379 (1.13%)*	*6 (1.58%)*	*2127 (1.18%)*
4	*8 (1.57%)*	*1585 (1.57%)*	*10 (1.94%)*	*2692 (1.50%)*
6	*3 (0.78%)*	*1241 (1.74%)*	*12 (2.44%)*	*2249 (1.70%)*
8	*6 (1.97%)*	*905 (2.24%)*	*6 (1.92%)*	*1632 (2.08%)*
10	*8 (3.08%)*	*675 (2.74%)*	*11 (5.24%)*	*1166 (2.50%)*
12	*8 (5.13%)*	*445 (3.08%)*	*5 (3.47%)*	*677 (2.52%)*
14	*1 (1.79%)*	*359 (3.86%)*	*1 (1.79%)*	*518 (2.96%)*
**No. (%) missing platelets[3]**				
2	*6 (1.59%)*	*1951 (1.60%)*	*10 (2.63%)*	*3075 (1.71%)*
4	*10 (1.97%)*	*2115 (2.09%)*	*23 (4.46%)*	*3980 (2.22%)*
6	*17 (4.43%)*	*1684 (2.36%)*	*10 (2.03%)*	*3180 (2.41%)*

8	*8 (2.63%)*	*1143 (2.82%)*	*19 (6.09%)*	*2064 (2.64%)*
10	*2 (0.77%)*	*700 (2.84%)*	*1 (0.48%)*	*1387 (2.98%)*
12	*16 (10.26%)*	*464 (3.21%)*	*14 (9.72%)*	*729 (2.71%)*
14	*1 (1.79%)*	*323 (3.47%)*	*3 (5.36%)*	*560 (3.21%)*
**Median time (range)[4]**				
2	*1.3 (0.0, 4.9)*	*1.6 (0.0, 5.0)*	*1.1 (0.0, 5.0)*	*1.6 (0.0, 5.0)*
4	*3.1 (0.2, 5.0)*	*3.4 (0.0, 5.0)*	*2.8 (0.3, 5.0)*	*3.5 (0.0, 5.0)*
6	*3.7 (0.4, 5.0)*	*4.2 (0.1, 5.0)*	*3.6 (0.7, 5.0)*	*4.2 (0.0, 5.0)*
8	*4.3 (1.2, 5.0)*	*4.4 (0.3, 5.0)*	*4.3 (1.8, 4.9)*	*4.4 (0.3, 5.0)*
10	*4.4 (1.5, 5.0)*	*4.5 (0.2, 5.0)*	*4.6 (2.1, 5.0)*	*4.5 (0.2, 5.0)*
12	*4.2 (2.9, 4.9)*	*4.6 (0.8, 5.0)*	*4.3 (2.5, 4.9)*	*4.5 (0.5, 5.0)*
14	*4.7 (3.7, 4.9)*	*4.6 (1.0, 5.0)*	*4.7 (3.9, 4.9)*	*4.6 (0.8, 5.0)*
**Median follow-up (range)[5]**				
2	*3.3 (1.8, 7.1)*	*3.6 (1.8, 7.2)*	*3.2 (1.8, 7.2)*	*3.6 (1.8, 7.2)*
4	*5.1 (2.1, 7.0)*	*5.4 (1.9, 7.2)*	*4.8 (2.1, 7.1)*	*5.5 (1.8, 7.2)*
6	*5.6 (2.3, 7.1)*	*6.2 (2.0, 7.2)*	*5.5 (2.4, 7.2)*	*6.1 (1.9, 7.2)*
8	*6.3 (2.9, 7.0)*	*6.4 (2.2, 7.2)*	*6.3 (3.6, 6.9)*	*6.4 (2.2, 7.2)*
10	*6.2 (3.6, 7.0)*	*6.5 (2.2, 7.2)*	*6.7 (3.9, 7.1)*	*6.5 (2.2, 7.2)*
12	*6.3 (4.7, 6.9)*	*6.5 (2.6, 7.2)*	*6.3 (4.4, 7.0)*	*6.5 (2.2, 7.2)*
14	*6.5 (5.6, 6.8)*	*6.6 (2.8, 7.2)*	*6.5 (5.8, 7.1)*	*6.6 (2.6, 7.2)*

[1]Patients with 1, 3, 5, etc. FBCs were available. However, descriptions here are limited to 2, 4, 6, etc. to highlight the general trend in summary statistics as the number of FBCs available changes.
[2]Age (years) at baseline FBC (most recent FBC available prior to two years before diagnosis/censor).
[3]Percentages are out of total number of FBCs in that age group.
[4]Time (years) between the first and last FBC in the five-year longitudinal period.
[5]Follow-up per patient is the time (years) from first FBC in the longitudinal period to diagnosis/censor.
Abbreviations: FBC=full blood count; Hb=haemoglobin; MCV=mean corpuscular volume.

Figure S3: C-statistic for the joint models and ColonFlag by number of FBCs in males (top), females (middle), and males aged 70-89 years at baseline FBC (bottom) (validation cohort)

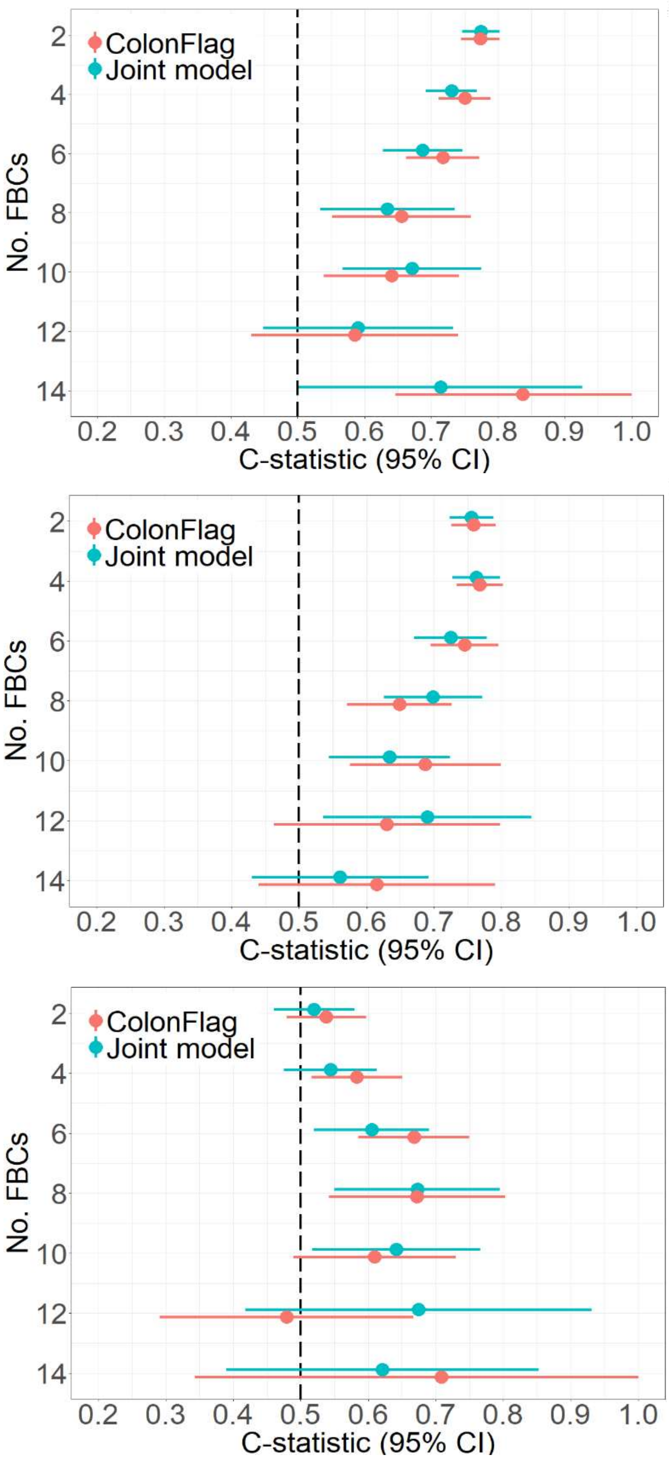

Number	Cases	Model	C-statistic (95%)
61200	189	Joint model	0.775 (0.746, 0.803)
		ColonFlag	0.774 (0.745, 0.802)
25427	127	Joint model	0.730 (0.692, 0.769)
		ColonFlag	0.750 (0.711, 0.789)
11971	64	Joint model	0.687 (0.628, 0.747)
		ColonFlag	0.717 (0.662, 0.772)
5099	38	Joint model	0.634 (0.534, 0.735)
		ColonFlag	0.655 (0.552, 0.759)
2491	26	Joint model	0.671 (0.568, 0.775)
		ColonFlag	0.640 (0.539, 0.741)
1216	13	Joint model	0.591 (0.448, 0.733)
		ColonFlag	0.586 (0.430, 0.741)
669	4	Joint model	0.714 (0.502, 0.926)
		ColonFlag	0.837 (0.647, 1.000)

Number	Cases	Model	C-statistic (95%)
90185	190	Joint model	0.755 (0.723, 0.788)
		ColonFlag	0.759 (0.726, 0.792)
44981	129	Joint model	0.763 (0.727, 0.799)
		ColonFlag	0.768 (0.734, 0.802)
22115	82	Joint model	0.725 (0.671, 0.779)
		ColonFlag	0.746 (0.695, 0.796)
9830	39	Joint model	0.699 (0.626, 0.772)
		ColonFlag	0.649 (0.572, 0.727)
4676	21	Joint model	0.634 (0.544, 0.724)
		ColonFlag	0.687 (0.576, 0.799)
2251	12	Joint model	0.690 (0.536, 0.844)
		ColonFlag	0.630 (0.462, 0.799)
1252	4	Joint model	0.561 (0.430, 0.692)
		ColonFlag	0.615 (0.440, 0.790)

Number	Cases	Model	C-statistic (95%)
11375	108	Joint model	0.538 (0.479, 0.597)
		ColonFlag	0.520 (0.460, 0.580)
7832	82	Joint model	0.583 (0.516, 0.651)
		ColonFlag	0.544 (0.475, 0.613)
4970	46	Joint model	0.668 (0.586, 0.749)
		ColonFlag	0.605 (0.520, 0.690)
2394	24	Joint model	0.672 (0.542, 0.803)
		ColonFlag	0.673 (0.550, 0.795)
1276	21	Joint model	0.609 (0.489, 0.729)
		ColonFlag	0.642 (0.517, 0.766)
614	6	Joint model	0.479 (0.291, 0.667)
		ColonFlag	0.675 (0.418, 0.931)
340	3	Joint model	0.709 (0.343, 1.000)
		ColonFlag	0.621 (0.390, 0.853)

Abbreviations: FBC=full blood count.

Figure S4: Calibration plots for the joint models by number of FBCs in males (top left) and females (top right) in the validation cohort and males in the development cohort (bottom left)

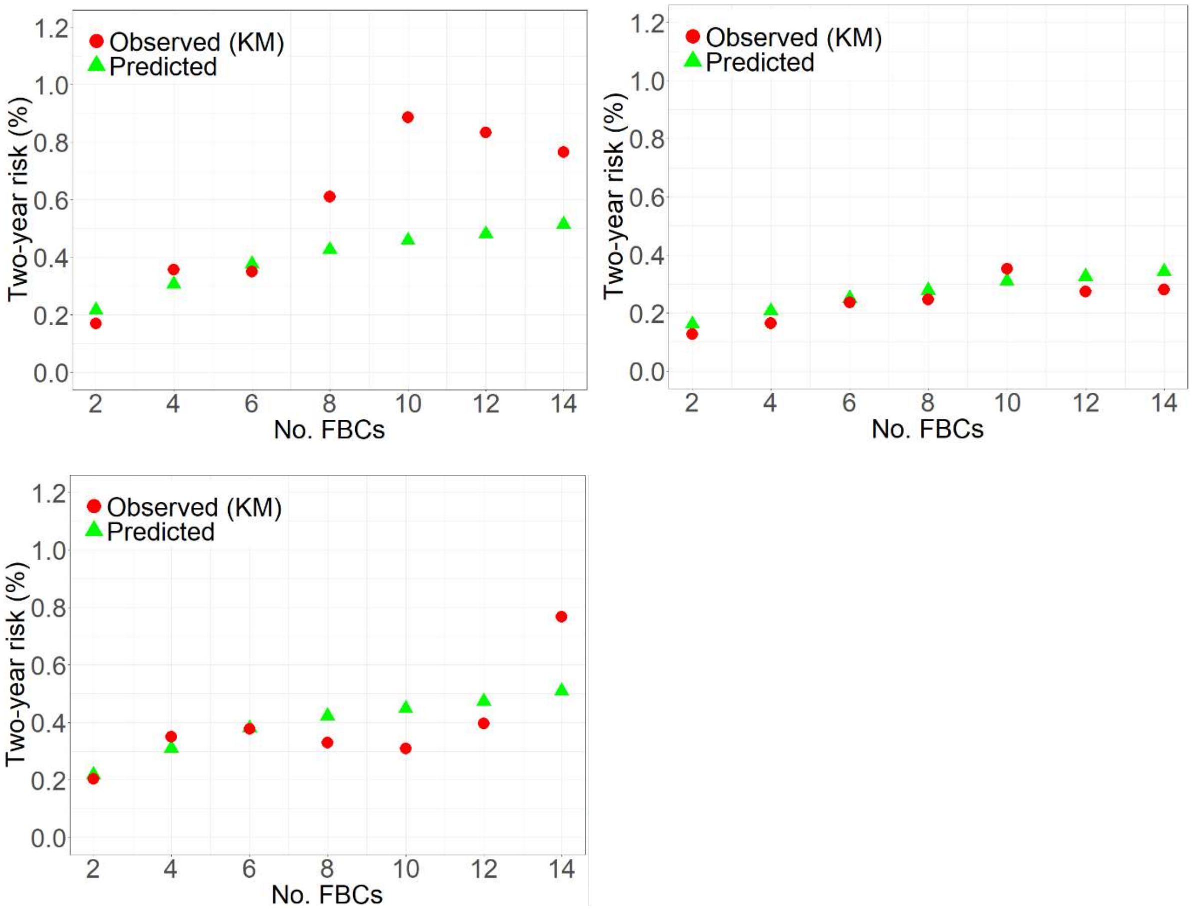

Abbreviations: KM=Kaplan-Meier; FBC=full blood count.

Table S8: Summary of patient characteristics and FBC data by time span on FBCs (validation cohort)

	Males		Females	
	**Diagnosed (1,040)**	**Not diagnosed (311,404)**	**Diagnosed (1,200)**	**Not diagnosed (461,700)**
**No. (%) patients per group**				
0≤ time <0.5	*280 (0.2%)*	*115,217 (99.8%)*	*295 (0.2%)*	*144,012 (99.8%)*
0.5≤ time <1.0	*49 (0.4%)*	*12,678 (99.6%)*	*78 (0.4%)*	*20,580 (99.6%)*
1.0≤ time <1.5	*67 (0.5%)*	*14,758 (99.6%)*	*79 (0.3%)*	*22,512 (99.7%)*
1.5≤ time <2.0	*77 (0.5%)*	*14,355 (99.5%)*	*68 (0.3%)*	*22,997 (99.7%)*
2.0≤ time <2.5	*77 (0.5%)*	*15,498 (99.5%)*	*84 (0.3%)*	*24,513 (99.7%)*
2.5≤ time <3.0	*79 (0.5%)*	*15,743 (99.5%)*	*104 (0.4%)*	*25,853 (99.6%)*
3.0≤ time <3.5	*74 (0.4%)*	*17,422 (99.6%)*	*91 (0.3%)*	*29,200 (99.7%)*
3.5≤ time <4.0	*96 (0.5%)*	*19,713 (99.5%)*	*95 (0.3%)*	*34,140 (99.7%)*
4.0≤ time <4.5	*117 (0.5%)*	*25,761 (99.6%)*	*136 (0.3%)*	*44,176 (99.7%)*
4.5≤ time <5.0	*120 (0.4%)*	*31,750 (99.6%)*	*169 (0.3%)*	*58,599 (99.7%)*
time ≥5.0	*4 (0.6%)*	*611 (99.4%)*	*1 (0.1%)*	*1,166 (99.9%)*
**Mean age (SD)[1]**				
0≤ time <0.5	*70.0 (10.7)*	*55.0 (12.1)*	*70.5 (12.5)*	*56.7 (14.2)*
0.5≤ time <1.0	*70.3 (9.4)*	*59.8 (13.3)*	*70.6 (13.4)*	*59.9 (15.5)*
1.0≤ time <1.5	*71.0 (11.0)*	*60.1 (13.0)*	*72.9 (12.1)*	*60.9 (15.0)*
1.5≤ time <2.0	*73.8 (10.7)*	*60.5 (12.7)*	*74.0 (10.6)*	*61.0 (14.5)*
2.0≤ time <2.5	*72.2 (10.8)*	*61.1 (12.4)*	*76.0 (9.1)*	*61.7 (14.5)*
2.5≤ time <3.0	*72.6 (8.4)*	*61.8 (12.2)*	*74.4 (9.4)*	*62.2 (14.1)*
3.0≤ time <3.5	*71.8 (10.3)*	*62.7 (12.1)*	*73.8 (10.1)*	*63.3 (13.9)*
3.5≤ time <4.0	*74.1 (9.6)*	*63.8 (11.9)*	*75.7 (11.4)*	*64.2 (13.7)*
4.0≤ time <4.5	*71.7 (9.4)*	*65.4 (11.8)*	*75.7 (10.5)*	*65.5 (13.5)*
4.5≤ time <5.0	*71.5 (10.1)*	*66.4 (11.7)*	*74.7 (9.6)*	*66.6 (13.3)*
time ≥5.0	*76.8 (4.6)*	*67.5 (11.2)*	*69.0 (-)*	*67.5 (12.9)*
**No. FBCs in total**				
0≤ time <0.5	*368*	*132,203*	*372*	*170,682*
0.5≤ time <1.0	*128*	*31,837*	*246*	*52,823*
1.0≤ time <1.5	*195*	*40,238*	*253*	*63,941*
1.5≤ time <2.0	*302*	*44,174*	*242*	*73,210*
2.0≤ time <2.5	*359*	*52,281*	*380*	*86,482*
2.5≤ time <3.0	*360*	*59,540*	*522*	*101,705*
3.0≤ time <3.5	*304*	*72,167*	*496*	*127,400*
3.5≤ time <4.0	*545*	*92,749*	*642*	*168,380*
4.0≤ time <4.5	*757*	*140,599*	*1079*	*250,589*
4.5≤ time <5.0	*1152*	*234,848*	*2079*	*473,410*
time ≥5.0	*43*	*6,725*	*59*	*15,628*
**Median no. FBCs (range)**				
0≤ time <0.5	*1 (1, 6)*	*1 (1, 16)*	*1 (1, 5)*	*1 (1, 13)*
0.5≤ time <1.0	*2 (1, 7)*	*2 (1, 19)*	*2 (1, 11)*	*2 (1, 29)*
1.0≤ time <1.5	*2 (1, 8)*	*2 (1, 26)*	*2 (1, 9)*	*2 (1, 28)*
1.5≤ time <2.0	*2 (1, 15)*	*2 (1, 45)*	*2 (1, 9)*	*2 (1, 42)*
2.0≤ time <2.5	*3 (1, 18)*	*2 (1, 85)*	*3 (1, 27)*	*2 (1, 55)*
2.5≤ time <3.0	*3 (1, 12)*	*2 (1, 100)*	*3 (1, 15)*	*2 (1, 71)*
3.0≤ time <3.5	*3 (1, 18)*	*3 (1, 71)*	*3 (1, 20)*	*3 (1, 78)*
3.5≤ time <4.0	*3 (1, 21)*	*3 (1, 67)*	*4 (1, 79)*	*3 (1, 69)*

4.0≤ time <4.5	*4 (1, 48)*	*3 (1, 89)*	*5 (1, 48)*	*3 (1, 90)*
4.5≤ time <5.0	*6 (1, 58)*	*5 (1, 141)*	*8 (1, 71)*	*5 (1, 134)*
time ≥5.0	*8 (1, 29)*	*7 (1, 105)*	*30 (1, 59)*	*10 (1, 219)*
**No. (%) missing Hb[2]**				
0≤ time <0.5	*2 (0.54%)*	*79 (0.06%)*	*0*	*119 (0.07%)*
0.5≤ time <1.0	*1 (0.78%)*	*96 (0.30%)*	*4 (1.63%)*	*178 (0.34%)*
1.0≤ time <1.5	*0*	*199 (0.49%)*	*1 (0.40%)*	*254 (0.40%)*
1.5≤ time <2.0	*1 (0.33%)*	*248 (0.56%)*	*9 (3.72%)*	*401 (0.55%)*
2.0≤ time <2.5	*2 (0.56%)*	*296 (0.57%)*	*4 (1.05%)*	*472 (0.55%)*
2.5≤ time <3.0	*0*	*375 (0.63%)*	*1 (0.19%)*	*654 (0.64%)*
3.0≤ time <3.5	*7 (2.30%)*	*479 (0.66%)*	*8 (1.61%)*	*718 (0.56%)*
3.5≤ time <4.0	*5 (0.92%)*	*528 (0.57%)*	*5 (0.78%)*	*949 (0.56%)*
4.0≤ time <4.5	*4 (0.53%)*	*886 (0.63%)*	*1 (0.09%)*	*1414 (0.56%)*
4.5≤ time <5.0	*11 (0.95%)*	*1248 (0.53%)*	*18 (0.87%)*	*2571 (0.54%)*
time ≥5.0	*0*	*17 (0.25%)*	*0*	*58 (0.37%)*
**No. (%) missing MCV[2]**				
0≤ time <0.5	*9 (2.45%)*	*351 (0.27%)*	*3 (0.81%)*	*567 (0.33%)*
0.5≤ time <1.0	*2 (1.56%)*	*423 (1.33%)*	*4 (1.63%)*	*725 (1.37%)*
1.0≤ time <1.5	*9 (4.62%)*	*585 (1.45%)*	*7 (2.77%)*	*889 (1.39%)*
1.5≤ time <2.0	*7 (2.32%)*	*788 (1.78%)*	*9 (3.72%)*	*1177 (1.61%)*
2.0≤ time <2.5	*5 (1.39%)*	*904 (1.73%)*	*8 (2.11%)*	*1393 (1.61%)*
2.5≤ time <3.0	*4 (1.11%)*	*1051 (1.77%)*	*22 (4.21%)*	*1783 (1.75%)*
3.0≤ time <3.5	*2 (0.66%)*	*1333 (1.85%)*	*15 (3.02%)*	*2194 (1.72%)*
3.5≤ time <4.0	*21 (3.85%)*	*1767 (1.91%)*	*25 (3.89%)*	*3039 (1.80%)*
4.0≤ time <4.5	*14 (1.85%)*	*2622 (1.86%)*	*60 (5.56%)*	*4670 (1.86%)*
4.5≤ time <5.0	*31 (2.69%)*	*5311 (2.26%)*	*56 (2.69%)*	*9623 (2.03%)*
time ≥5.0	*0*	*136 (2.02%)*	*0*	*295 (1.89%)*
**No. (%) missing platelets[2]**				
0≤ time <0.5	*3 (0.82%)*	*394 (0.30%)*	*0 (0.00%)*	*638 (0.37%)*
0.5≤ time <1.0	*3 (2.34%)*	*447 (1.40%)*	*9 (3.66%)*	*832 (1.58%)*
1.0≤ time <1.5	*9 (4.62%)*	*640 (1.59%)*	*5 (1.98%)*	*1064 (1.66%)*
1.5≤ time <2.0	*8 (2.65%)*	*931 (2.11%)*	*16 (6.61%)*	*1391 (1.90%)*
2.0≤ time <2.5	*4 (1.11%)*	*1103 (2.11%)*	*16 (4.21%)*	*1885 (2.18%)*
2.5≤ time <3.0	*3 (0.83%)*	*1323 (2.22%)*	*24 (4.60%)*	*2383 (2.34%)*
3.0≤ time <3.5	*11 (3.62%)*	*1703 (2.36%)*	*8 (1.61%)*	*3059 (2.40%)*
3.5≤ time <4.0	*22 (4.04%)*	*2155 (2.32%)*	*15 (2.34%)*	*4095 (2.43%)*
4.0≤ time <4.5	*8 (1.06%)*	*3171 (2.26%)*	*70 (6.49%)*	*5982 (2.39%)*
4.5≤ time <5.0	*43 (3.73%)*	*5594 (2.38%)*	*66 (3.17%)*	*11018 (2.33%)*
time ≥5.0	*5 (11.63%)*	*161 (2.39%)*	*0 (0.00%)*	*244 (1.56%)*

[1]Age (years) at baseline FBC (most recent FBC available prior to two years before diagnosis/censor).
[2]Percentages are out of total number of FBCs in that age group.
Abbreviations: FBC=full blood count; Hb=haemoglobin; MCV=mean corpuscular volume.

Figure S5: C-statistic for the joint models and ColonFlag by time span of FBCs in males (top) and females (bottom) (validation cohort)

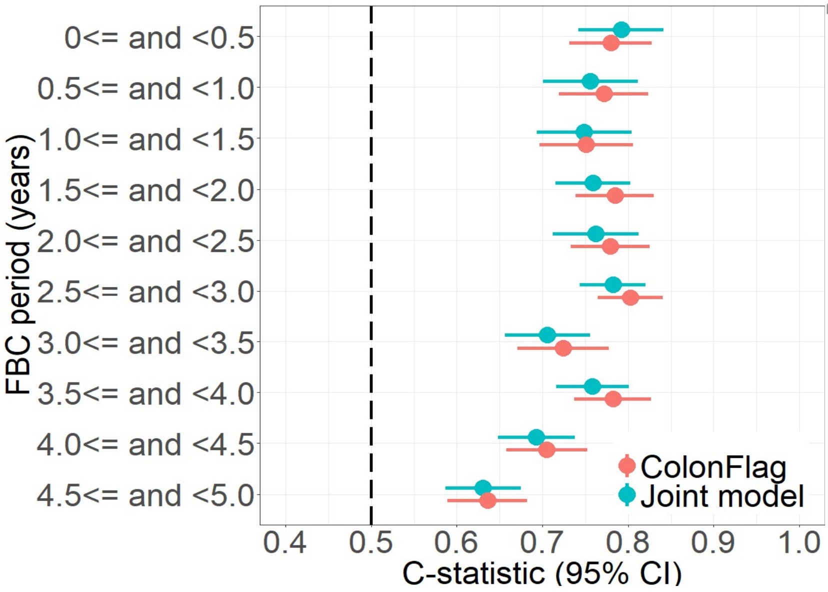

Number	Cases	Model	C-statistic (95% CI)
13599	58		0.780 (0.731, 0.828)
			0.792 (0.742, 0.842)
12727	49		0.771 (0.719, 0.824)
			0.756 (0.700, 0.812)
14825	67		0.751 (0.697, 0.805)
			0.749 (0.693, 0.804)
14432	77		0.785 (0.739, 0.830)
			0.759 (0.715, 0.803)
15575	77		0.779 (0.733, 0.825)
			0.762 (0.712, 0.812)
15822	79		0.803 (0.765, 0.840)
			0.782 (0.744, 0.820)
17496	74		0.724 (0.671, 0.778)
			0.706 (0.656, 0.755)
19809	96		0.782 (0.737, 0.827)
			0.759 (0.716, 0.801)
25878	117		0.705 (0.657, 0.752)
			0.693 (0.648, 0.738)
31870	120		0.636 (0.589, 0.682)
			0.631 (0.587, 0.675)

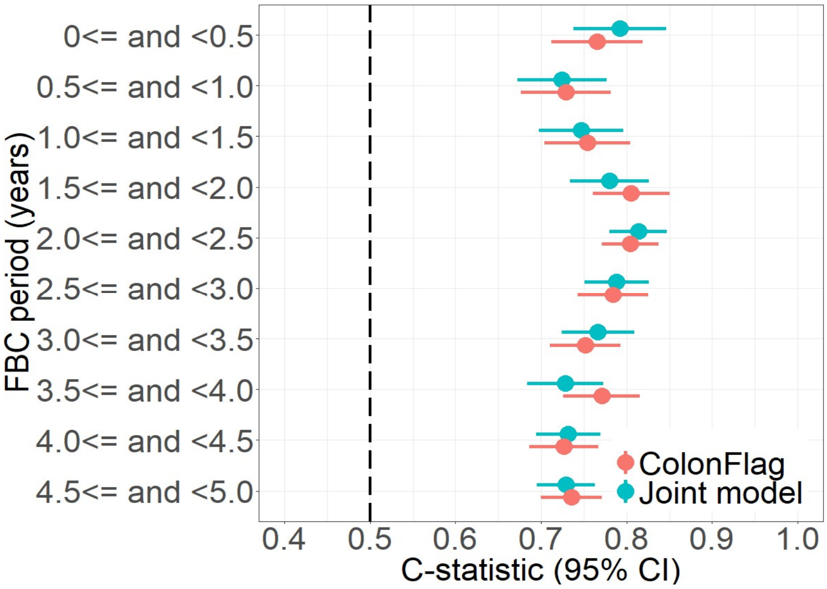

Number	Cases	Model	C-statistic (95% CI)
21065	59		0.792 (0.738, 0.847)
			0.765 (0.712, 0.819)
20658	78		0.724 (0.672, 0.777)
			0.729 (0.676, 0.781)
22591	79		0.747 (0.698, 0.796)
			0.754 (0.704, 0.804)
23065	68		0.780 (0.734, 0.826)
			0.805 (0.761, 0.850)
24597	84		0.814 (0.780, 0.847)
			0.804 (0.771, 0.837)
25957	104		0.788 (0.751, 0.826)
			0.784 (0.743, 0.825)
29291	91		0.766 (0.724, 0.809)
			0.752 (0.711, 0.793)
34235	95		0.728 (0.683, 0.773)
			0.771 (0.726, 0.815)
44312	136		0.732 (0.694, 0.769)
			0.727 (0.686, 0.767)
58768	169		0.729 (0.695, 0.763)
			0.736 (0.700, 0.771)

Abbreviations: FBC=full blood count.

Figure S6: Calibration plots for the joint models by time span of FBCs in males (left) and females (right) (validation cohort)

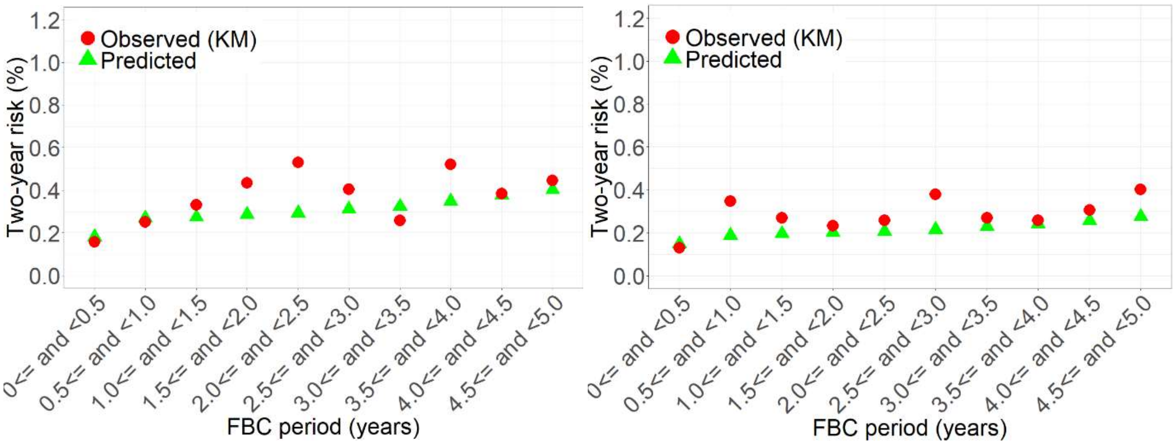

Abbreviations: KM=Kaplan-Meier; FBC=full blood count.
